# Glycosyltransferase B4GALNT1 promotes immunosuppression in hepatocellular carcinoma via the HES4-SPP1-TAM/Th2 axis

**DOI:** 10.1186/s43556-024-00231-w

**Published:** 2024-12-01

**Authors:** Zhifeng Wang, Jiaxin Liu, Xiaoming Wang, Qingyun Wu, Qiao Peng, Tianxiao Yang, Xuehui Sun, Xiaofeng Wang, Yilin Wang, Weicheng Wu

**Affiliations:** 1 Human Phenome Institute, State Key Laboratory of Genetic Engineering and MOE Key Laboratory of Contemporary Anthropology, School of Life Sciences, Fudan University, Shanghai, China; 2 Department of Hepatic Surgery, Department of Oncology, Shanghai Medical College, Fudan University Shanghai Cancer Center, Fudan University, Shanghai, China; 3 Department of Hepatobiliary Surgery, The First Affiliated Hospital of Wannan Medical College, Wuhu, China; 4 Fudan University-the People’s Hospital of Rugao Joint Research Institute of Longevity and Ageing, Rugao, Jiangsu China; 5 Shanghai Tenth People’s Hospital, School of Medicine, Tongji University Cancer Center, Tongji University, Shanghai, China

**Keywords:** Hepatocellular carcinoma, B4GALNT1, Prognosis, Immunosuppression

## Abstract

β-1,4-N-acetylgalactosaminyltransferase I (B4GALNT1) is a key glycosyltransferase for gangliosides. Its aberrant expression has been observed in various cancers, and its potential roles in tumor immunity were suggested recently. However, how B4GALNT1 regulate tumor progression and tumor immunity remains largely unknown. In this study, we aimed to investigate the roles of B4GALNT1 in hepatocellular carcinoma (HCC), particularly in reshaping the tumor immune microenvironment, and evaluate the potential beneficial effects of targeting B4GALNT1 in immunotherapy. Our data verified the aberrant upregulation of B4GALNT1 in HCC tumor tissues and tumor cells, which could be utilized as an independent prognostic factor and improve the predicting performance of traditional tumor node metastasis (TNM) system. We also demonstrated that B4GALNT1 increased the phosphorylation of Hes Family BHLH Transcription Factor 4 (HES4) via p38 mitogen-activated protein kinase (p38)/ c-Jun N-terminal kinase (JNK) signaling in tumor cells, thus increasing the transcriptional activity of HES4, which upregulated the synthesis and secretion of secreted phosphoprotein 1 (SPP1), modulated the composition of tumor-associated macrophages (TAMs) and T helper type 2 (Th2) cells, and eventually reshaped the immunosuppressive microenvironment. In addition, silencing B4GALNT1 was proved to enhance the tumor-killing efficiency of the programmed cell death protein 1 (PD-1)-targeting strategy in mouse model. In conclusion, this study evaluated B4GALNT1 as a prognostic predictor for HCC patients and revealed the mechanism of B4GALNT1 in microenvironmental remodeling, which extends the understanding of HCC progression and provides a novel auxiliary strategy for HCC immunotherapy.

**Supplementary Information:**

The online version contains supplementary material available at 10.1186/s43556-024-00231-w.

## Introduction

Hepatocellular carcinoma (HCC) continues to pose a daunting global health challenge, with its high mortality and limited treatment options for advanced stages reflected in a disheartening 5-year survival rate of approximately 18% [1]. The high incidence and mortality rates, corresponding to approximately 830,000 deaths per year, highlight the dire prognosis for those afflicted with this disease [2]. In the past two decades, the development of immunotherapy has significantly improved the prognosis of patients with advanced HCC [3]. The primary strategy in HCC immunotherapy involves targeting immune checkpoints, most notably the programmed cell death protein 1 (PD-1) and programmed death-ligand 1 (PD-L1) pathway. In addition, a variety of other immune regulatory molecules, including mucin molecules, lymphocyte activation genes, cytotoxic T-lymphocyte-associated protein 4 (CTLA-4), and lymphocyte attenuation factors, play crucial roles in modulating the immune response in HCC [3]. Despite these advances, monotherapy with immunotherapy faces several challenges, such as low response rates and the emergence of primary and acquired resistance. A prominent example of these limitations is primary resistance to PD-1/PD-L1 blockade, which often occurs due to insufficient PD-1 expression or a lack of immune cell infiltration within the tumor microenvironment (TIME). In these cases, the immune system cannot effectively recognize and target tumor cells, thus limiting the effectiveness of immune checkpoint inhibitors (ICBs) from the beginning [4]. Thus, there is a pressing demand to pioneer innovative immuno-targeted agents and delve deeper into the intricacies of immune regulation in HCC. Understanding these mechanisms is vital for overcoming the hurdles of primary and acquired resistance to immunotherapy and ultimately offers renewed hope to patients with advanced HCC.

As one of the factors of immune dysregulation, aberrant glycosylation is observed in various cancers, and many glycolipids undergo a wide range of glycosylation alterations, with implications for biological function during malignant transformation [5]. Among these glycolipids, gangliosides, which are glycosphingolipids containing neuraminic acid, have been extensively studied [6]. Glycosylation changes the amount and type of ganglioside in cancers, which results in the promotion of tumor proliferation and stemness, tumor cell invasion and metastasis, tumor angiogenesis, and tumor immune suppression [7, 8]. For example, increased N-acetylgalactosamine (GalNAc) modification elevates the levels of ganglioside GM2 and GD2 in the blood of patients with neuroblastoma, retinoblastoma, and HCC [9, 10], helping tumors escape the immune system by inducing apoptosis in T and natural killer cell (NK) cells, inhibiting the tumor-killing activity of various immune cells, and promoting the immunosuppressive effect of some immune cells [11, 12].

Alterations in the glycosylation of these gangliosides result from abnormal activation or aberrant expression of corresponding glycosyltransferases, and many glycosyltransferases have been shown to be associated with cancer progression [13–15]. β−1,4-N-acetylgalactosaminyltransferase I (B4GALNT1) is the key transferase involved in the ratio of GM2 to GD2 in different tumors. B4GALNT1 can transfer N-acetylgalactosamine (GalNAc) from uridine diphosphate-N-acetylgalactosamine (UDP-GalNAc) to GM3 or GD3 and then generate GM2 or GD2, respectively [16]. Considering the critical roles of GM2/GD2 in tumor progression, a growing body of research has focused on the roles of B4GALNT1 in cancers. High levels of B4GALNT1 have been detected in adult leukemia cells, neuroblastoma cell lines, glioma cell lines, and some malignant melanoma cell lines [17, 18]. B4GALNT1 upregulation has been observed in gastric cancer as well as several types of lung cancer [19, 20]. Recent pan-cancer studies also revealed the prognostic value of B4GALNT1 in multiple cancer types and discussed its potential effects on tumor immunity [21]. However, the immunological functions of B4GALNT1 remain largely unknown.

To explore the immunoregulatory mechanisms of B4GALNT1 in HCC, we conducted a series of comprehensive analyses on the expression patterns, prognostic implications, and functions of B4GALNT1 in HCC via both bioinformatic and experimental approaches. Our findings verified the prognostic value of upregulated B4GALNT1 in HCC patients and revealed the immunosuppressive functions of B4GALNT1, as well as its intrinsic regulatory effects on cell communication and transcriptional regulation. Notably, our xenograft model demonstrated that targeting B4GALNT1 not only inhibits tumor growth but also increases the efficacy of immunotherapeutic approaches. Our findings pioneer the investigation into the critical role of B4GALNT1 in HCC, especially in the context of tumor progression and immunosuppression, which advances the understanding of HCC pathogenesis and lays the groundwork for developing more precise and personalized treatment plans. This could lead to improved outcomes for patients and a more nuanced approach to HCC management, emphasizing the importance of multitarget immune therapies in the future of cancer treatment.

## Results

### B4GALNT1 is upregulated in HCC tumor cells

**Fig. 1:**
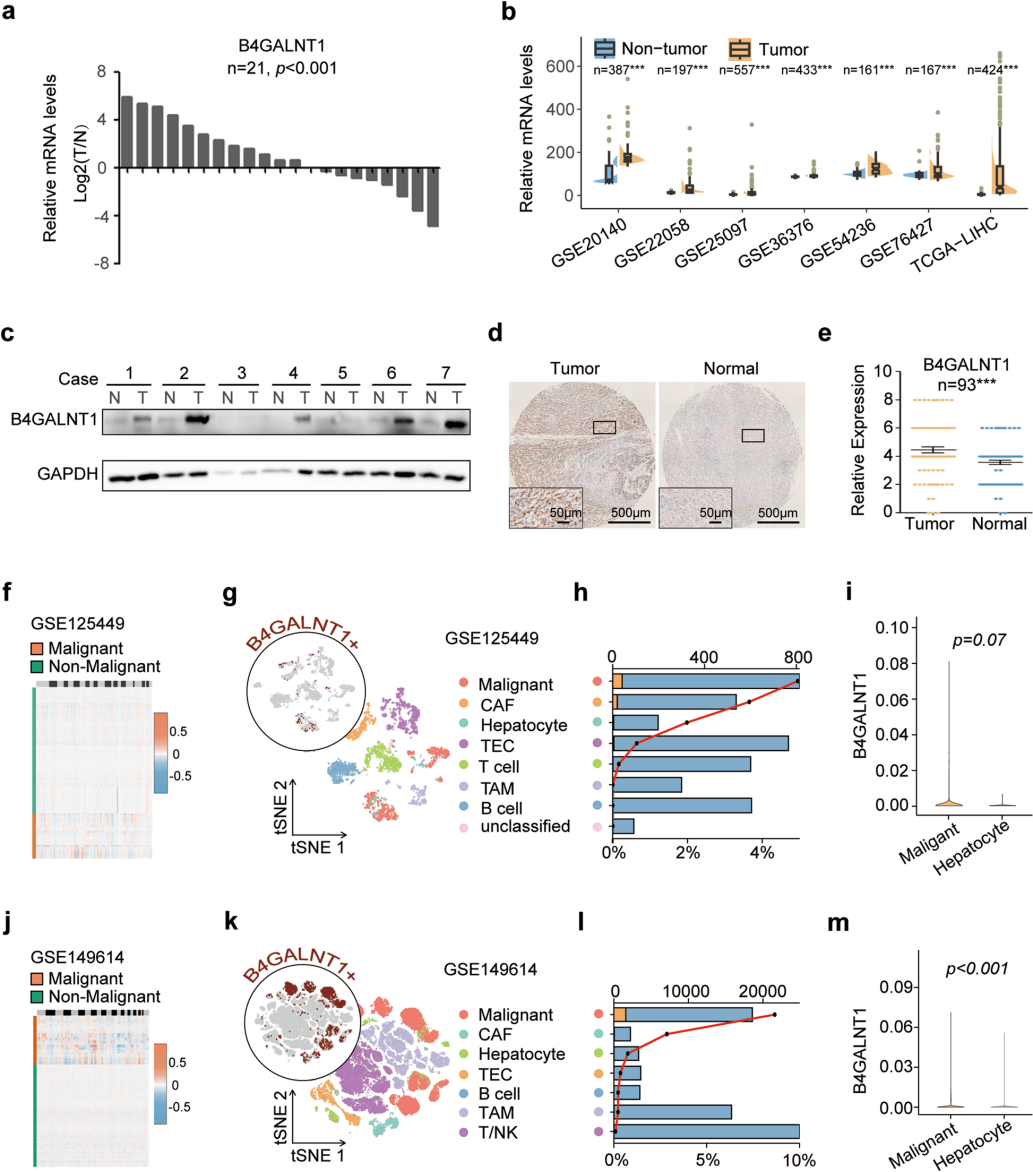
The upregulation of B4GALNT1 in HCC tumor tissues and tumor cells. **a** Relative mRNA expression of B4GALNT1 was determined by real-time PCR in 21 pairs of HCC tumor tissues and adjacent non-tumor tissues. **b** Relative mRNA expression levels of B4GALNT1 in HCC tissues and normal liver tissues obtained from TCGA-LIHC, GSE20140, GSE22058, GSE25097, GSE36376, GSE54236 and GSE76427 datasets. **c** Western blot analysis was applied to detect relative protein expression level of B4GALNT1 in 21 pairs of HCC tissues. GAPDH was used as control. Five representative pairs were showed. T, HCC tumor tissue; N, adjacent non-tumor tissue. **d** Representative IHC staining of B4GALNT1 in HCC tumor tissue and adjacent non-tumor tissue. Regional magnification image showed below. **e** Statistical data of the IHC analysis (*n* = 93). **f** Large-scale copy number variation (CNV) patterns of single cells (rows) of GSE125449 inferred from single-cell transcriptomes. Orange, Malignant; Green, non-Malignant. **g** t**-**SNE plot of cell types and B4GALNT1 expression distribution (indicated by brown) of GSE125449 database. **h** Numbers (bar plot, top x-axis) and proportions (line chart, bottom x-axis) of different cell types in tumor tissues from GSE125449 of B4GALNT1 positive cells in these clusters. Orange, B4GALNT1-positive (B4+) tumor cell; Blue, B4GALNT1-negative (B4-) tumor cell. **i** Expressional comparison of B4GALNT1 between malignant tumor cells and non-malignant hepatocytes in GSE125449 database. **j** Large-scale CNV patterns of single cells (rows) of GSE149614 inferred from single-cell transcriptomes. Orange, Malignant; Green, non-Malignant. **k** t**-**SNE plot of cell types and B4GALNT1 expression distribution (indicated by brown) of GSE149614 database. **l** Numbers (bar plot, top x-axis) and proportions (line chart, bottom x-axis) of different cell types in tumor tissues from GSE149614 of B4GALNT1 positive cells in these clusters. Orange, B4GALNT1-positive (B4+) tumor cell; Blue, B4GALNT1-negative (B4-) tumor cell. **m** Expressional comparison of B4GALNT1 between malignant tumor cells and non-malignant hepatocytes in GSE149614 database. Data are represented as the mean ± SD, Statistical analysis was performed using Student’s t-test, ***p < 0.001

Since the upregulation of B4GALNT1 in HCC tumor tissues has been previously reported [21], we first attempted to verify this aberrant upregulation in our collected samples and public datasets. We observed elevated mRNA levels of B4GALNT1 in tumor tissues from 21 Chinese patients with HCC (*p* < 0.001) (Fig. 1a), as well as the similar overexpression pattern at mRNA levels in several public HCC datasets, including The Cancer Genome Atlas Liver Hepatocellular Carcinoma (TCGA-LIHC), GSE20140, GSE22058, GSE25097, GSE36376, GSE54236, and GSE76427 (Fig. 1b). Increased protein levels of B4GALNT1 in tumor tissues were also observed in Western Blot (WB) tests on 21 patients (Fig. 1c and Fig. S1) and immunohistochemistry (IHC) assays on 93 other patients (*p* < 0.001) (Fig. 1d and e). These data demonstrated the upregulation of B4GALNT1 in HCC tumor tissues.

Although IHC images revealed that B4GALNT1 was expressed mainly in the cytoplasm of tumor cells, its positive staining in other cell types could also be observed (data not shown). To verify the major B4GALNT1-expressing cell type, we further analyzed the single-cell data of HCC tumor tissues from 9 patients (GSE125449) and 10 other patients (GSE149614). Via CopyKAT analysis, we confidently distinguished the malignant cells (Fig. 1f and j). And we found that malignant cells conferred highest percentage of B4GALNT1-positive cells among all cell types (Fig. 1g, h, k, and l), indicating the majority of B4GALNT1expression in tumor cells. Meanwhile, in GSE149614 dataset, B4GALNT1 levels were significantly higher in malignant cells than those in nonmalignant hepatocytes (*p* < 0.001, n^malignant^ = 18565, n^non−malignant^ =3315) (Fig. 1m). Although no significant difference was observed in GSE125449 (*p* = 0.07, n^malignant^ = 814, n^nonmalignant^ = 198) due to the small sample size of B4GALNT1-positive malignant cells, the average B4GALNT1 level of malignant cells was still greater than that of nonmalignant hepatocytes (Fig. 1i). Taken together, these findings demonstrate that B4GALNT1 is upregulated in HCC tumor tissues, and such upregulation predominantly occurred in malignant cells.

### The upregulation of B4GALNT1 predicts poor prognosis in HCC patients

**Fig. 2:**
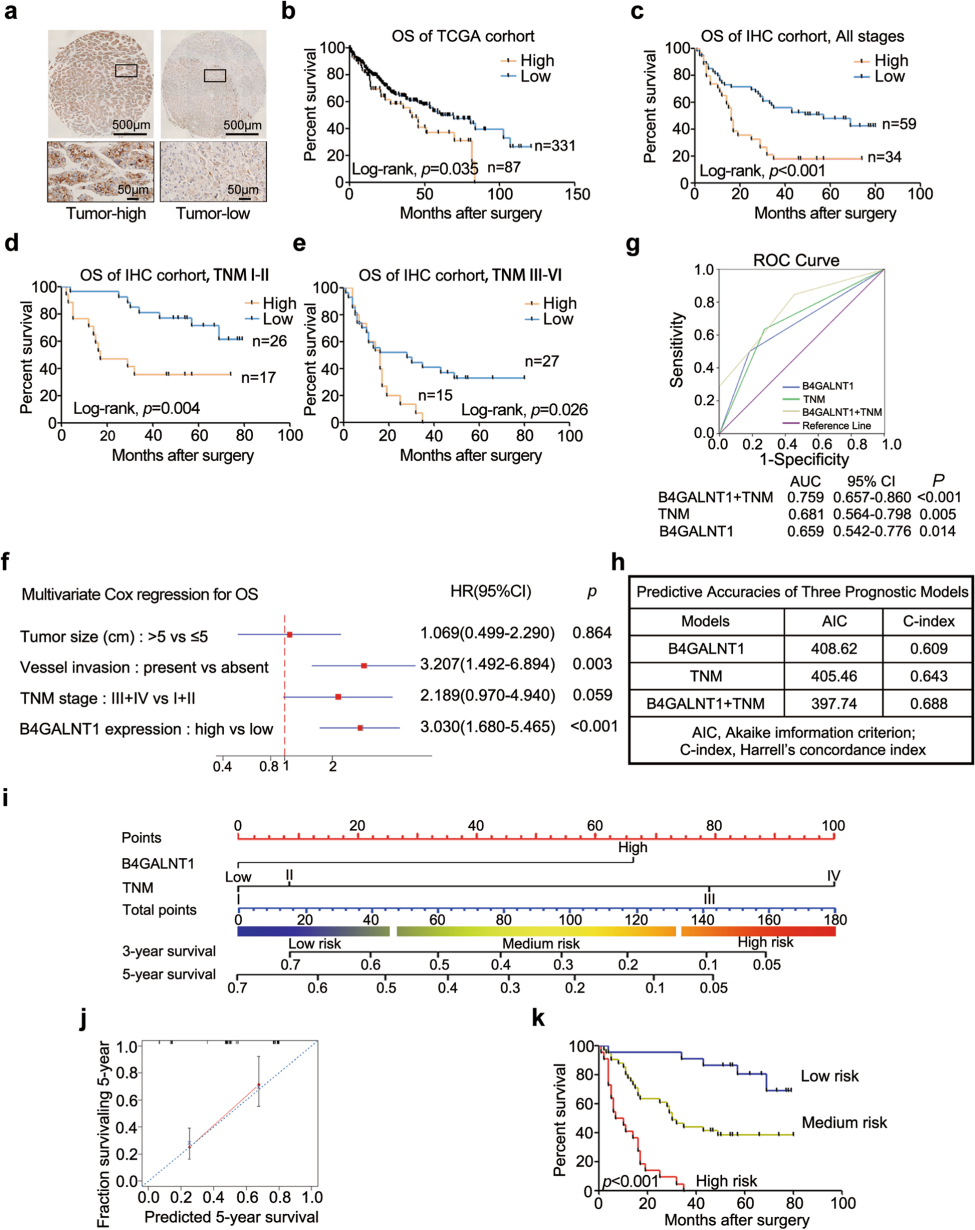
The prognostic value of B4GALNT1 in HCC. **a** The representative IHC staining of high and low expression level of B4GALNT1 in HCC tumor tissues. **b** Kaplan-Meier analysis for overall survival of HCC patients based on B4GALNT1 mRNA expression level obtained from TCGA-LIHC dataset. **c-e** Kaplan-Meier analysis for overall survival of HCC patients in all patients, in patients at TNM I-II stage, and in patients at TNM III-IV stage using our IHC staining score. **f** ROC curve analysis of the sensitivity and specificity for the predictive value of TNM model, B4GALNT1 expression, and the combination model. **g** Independent prognostic factors for overall survival of patients with HCC in IHC cohort identified by multivariate Cox regression analysis. **h** AIC and Harrell’s C-index analysis was used to compare the predictive accuracies of TNM model, B4GALNT1 expression, and the combination model. **i** Nomogram for predicting overall survival in patients with HCC was established by integrating TNM staging system and B4GALNT1 expression. **j** Calibration plot for predicting survival at 5 years which proved the good performance of the nomogram. **k** According to the total points in nomogram, patients were divided into three groups (low risk, medium risk, and high risk). Kaplan–Meier analysis was applied to determine the correlation between the risk and overall survival and verify the prognostic efficiency of nomogram

To investigate the effects of B4GALNT1 upregulation on HCC progression, we performed several clinicopathological analysis, and observed that the upregulation of B4GALNT1 was associated with advanced T stage (*p* = 0.022), TNM stage (*p* = 0.030) (Table S1), and worse overall survival in both the TCGA-LIHC cohort (*p* = 0.035) and our cohort (*p* < 0.001) (Fig. 2a and b). After dividing all patients into early (TNM I-II) and advanced (TNM III-IV) groups, we could still observe the association between elevated B4GALNT1 and shorter survival times in stratified subgroups (Fig. 2c, d and e). Moreover, both univariate and multivariate Cox regression analysis supported B4GALNT1 expression as an independent predictive factor for the overall survival of HCC patients (Fig. 2f and Table S2). And adding B4GALNT1 levels into the traditional outcome prediction model based on TNM staging could markedly improve the predictive ability of the raw TNM model, especially on the discrimination and calibration assessed with receiver operating characteristic (ROC) curve, Harrell’s concordance index (C-index) and the Akaike information criterion (AIC) (Fig. 2g and h). We also developed a predictive nomogram with the combination of B4GALNT1 levels and TNM staging (Fig. 2i and j), and validated its efficiency on discriminating the risk of HCC patients (Fig. 2k). Taken together, our data support the prognostic value of B4GALNT1 levels in HCC.

### B4GALNT1 is involved in immune regulation

**Fig. 3:**
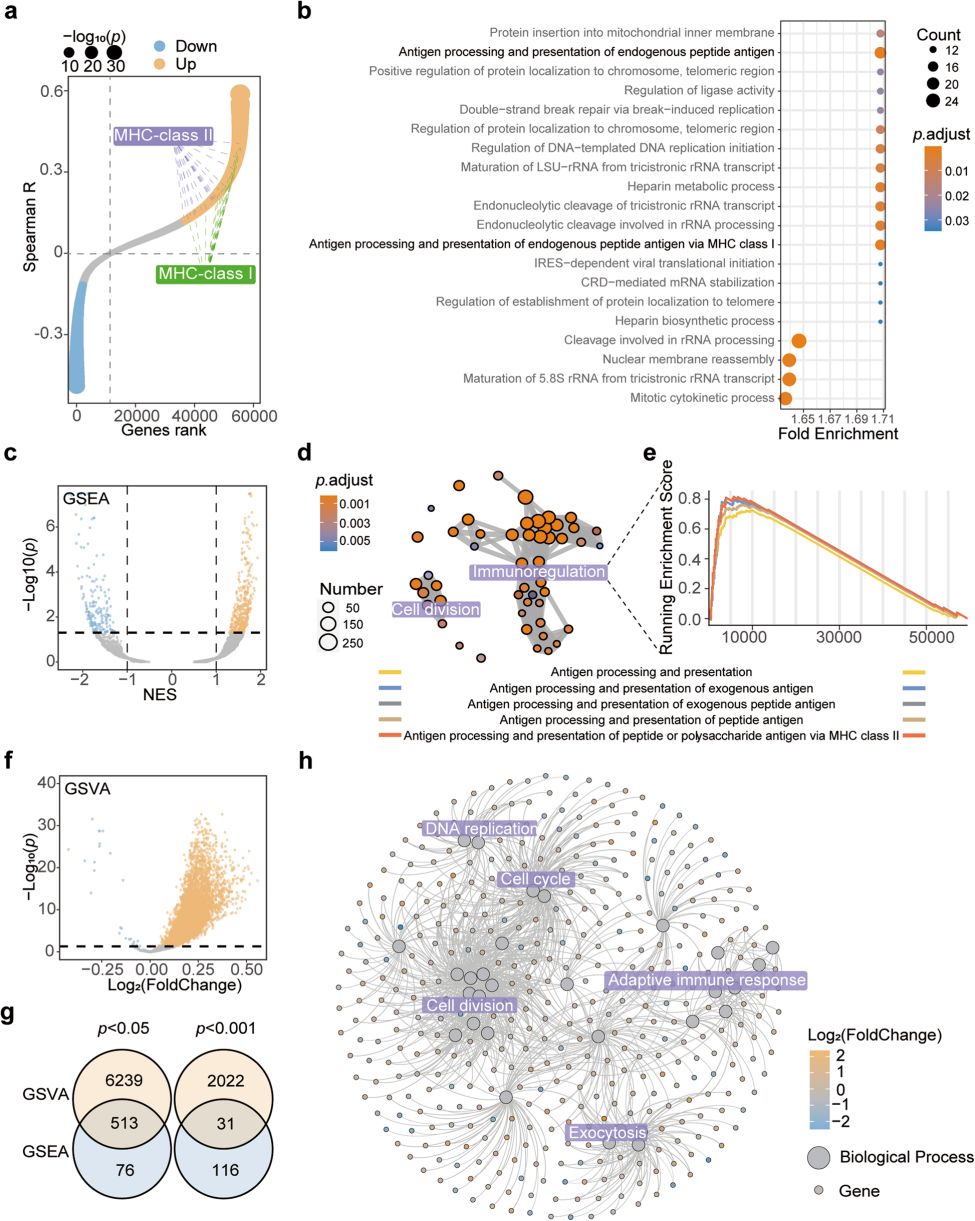
B4GALNT1-associated functions in HCC. **a** Expressional correlation analysis between B4GALNT1 and other genes in TCGA-LIHC. **b** GO enrichment for the genes significantly correlated with B4GALNT1. **c** GSEA analysis for the associated functions of B4GALNT1. **d** Network analysis for the major B4GALNT1-associated functions from GSEA results. **e** Top 5 GSEA functions associated with B4GALNT1. **f** GSVA analysis for B4GALNT1-associated functions. **g** Intersections between GEVA and GSEA results under *p* < 0.05 and *p* < 0.001. **h** Integration network analysis for 410 B4GALNT1-associated genes and 31 intersected pathways between GSVA and GSEA results (*p* < 0.001)

**Fig. 4:**
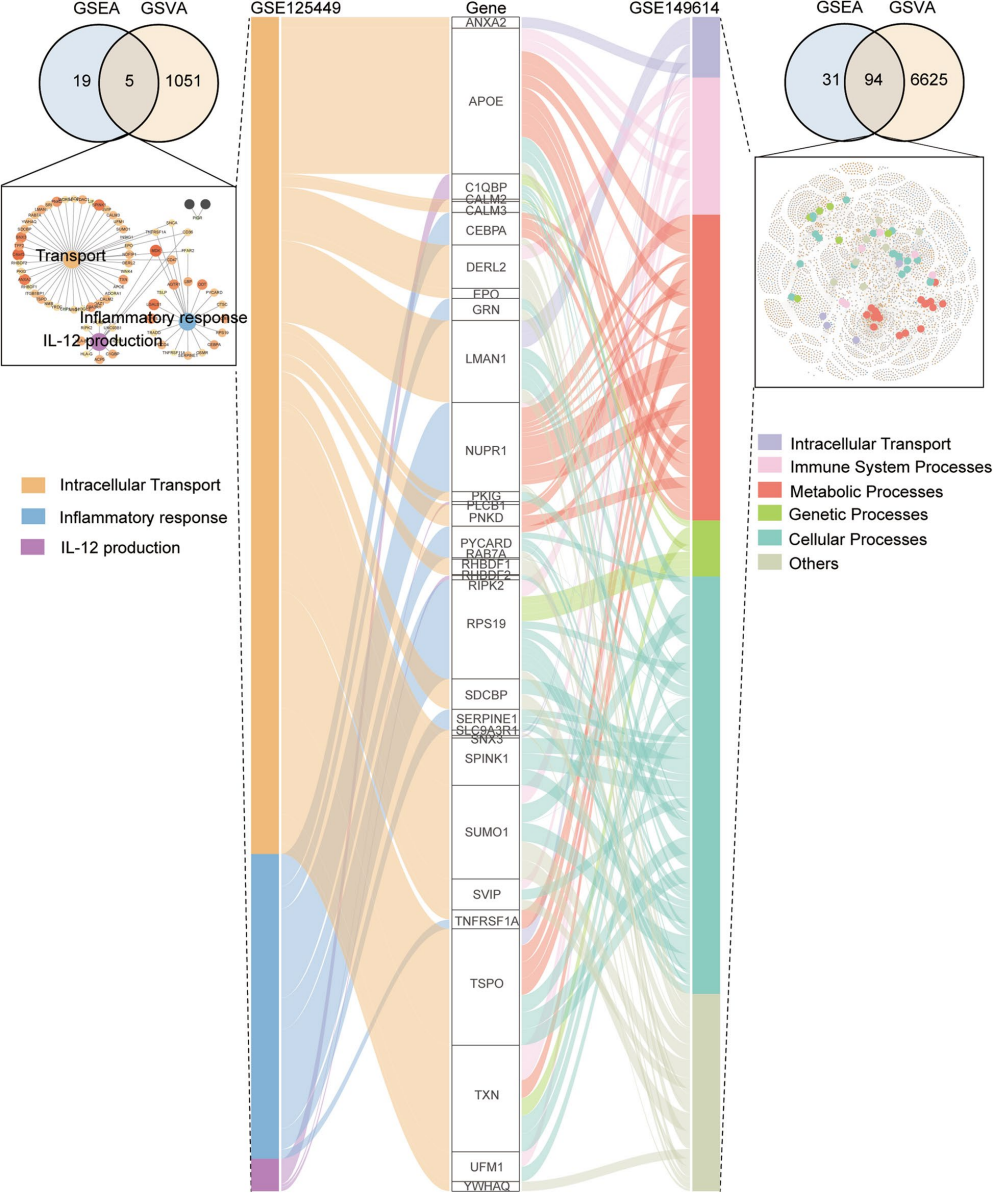
B4GALNT1-associated functions in HCC malignant cells from different single-cell data. B4GALNT1-associated GSEA and GSVA analysis were performed in the distinguished malignant cells from GSE125449 (left) and GSE149614 (right), and the intersected pathways for each dataset were further analysis for core network modules. Genes that were associated with these pathways and simultaneously correlated with B4GALNT1 were listed in Sankey plot (middle)

To further elucidate the biological effects of B4GALNT1, we performed correlation analysis on TCGA-LIHC data, and identified all B4GALNT1-correlated genes (Fig. 3a). The functional enrichment of these genes revealed that B4GALNT1 was closely associated with antigen presentation (Fig. 3b). Gene set enrichment analysis (GSEA) also revealed the roles of B4GALNT1 in immunoregulation (Fig. 3c and d), especially in the regulation of antigen processing and presentation (Fig. 3e). And gene set variation analysis (GSVA) got similar results with GSEA data on pathways related to antigen processing and presentation (Fig. 3f and g and Table S3). Under a stringent screening criteria (*p* < 0.001), 31 intersecting pathways between GSEA and GSVA results were screen out to construct an B4GALNT1-associated function network (Fig. 3g and h). In this network, immunoregulation was still one of the major hub functions (Fig. 3h). Moreover, B4GALNT1-associated pathways in malignant cells from single-cell RNA datasets included inflammatory response, intracellular transport, IL-12 production, and immune system processes (Fig. 4), supporting the roles of malignant cells-derived B4GALNT1 in immune regulation. In addition, the associated roles of B4GALNT1 in intracellular transport suggest that the effects of malignant cell-derived B4GALNT1 on tumor immunity might be achieved by the regulation of the transport or secretion of cytokines or chemokines.

### B4GALNT1 regulates the infiltration of immunosuppressive tumor-associated macrophages (TAMs) and T cells

**Fig. 5:**
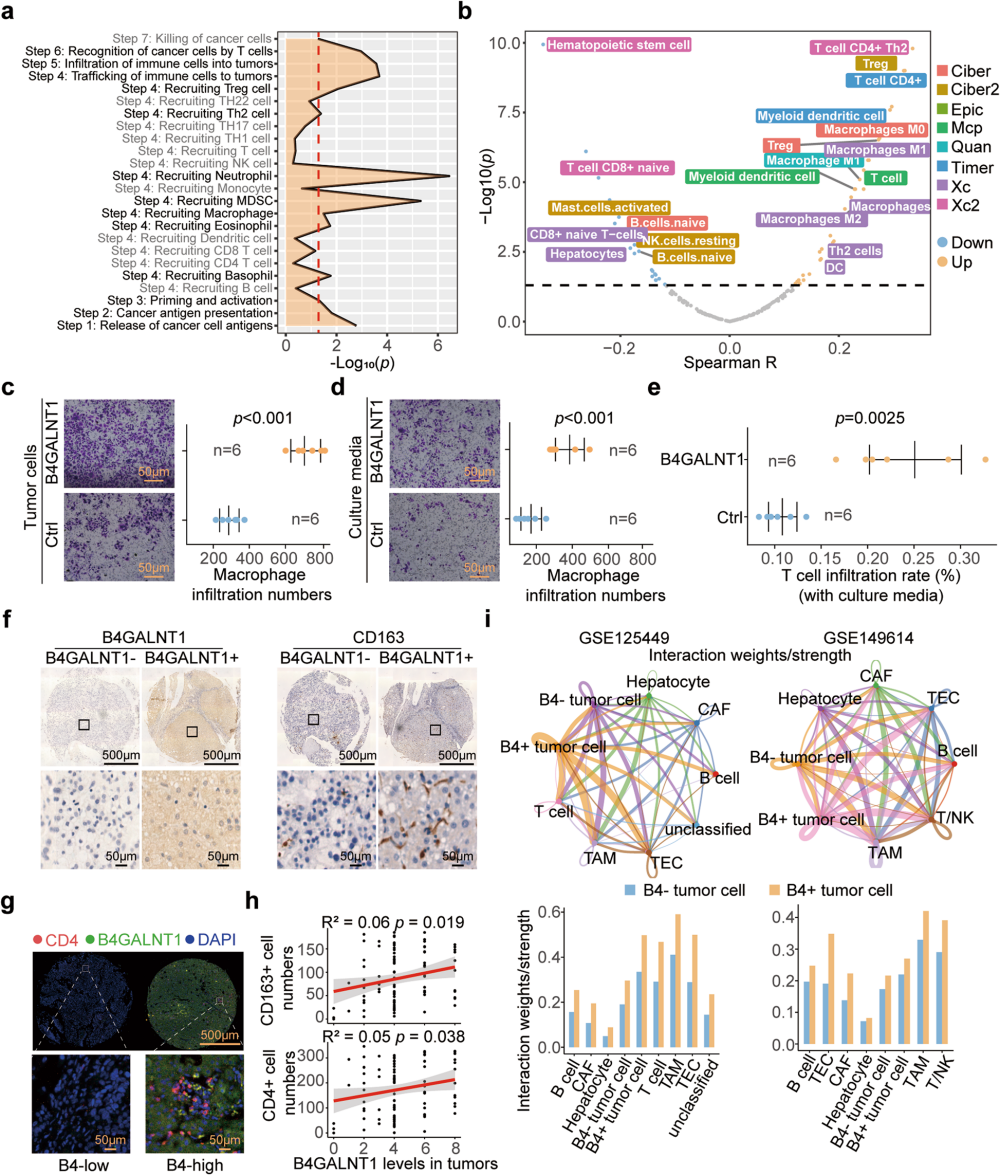
Immunosuppressive effects of B4GALNT1 in HCC. **a** B4GALNT1-associated immune steps analyzed by the comparison between B4GALNT1^high^ and B4GALNT1^low^ patients from TCGA-LIHC. **b** Correlations between estimated immune cell numbers and B4GALNT1 levels in HCC tumor tissues. **c**-**e** The recruitment of Macrophages (**c** and **d**) and T cells (**e**) assessed in transwell assays. Tumor cells with or without B4GALNT1 overexpression (**c**) or culture media of these cells (**d** and **e**) were added in the lower chamber under the transwell insert. **f** Representative IHC staining of B4GALNT1 and CD163-positive macrophages in B4GALNT1^high^ and B4GALNT1^low^ HCC tumor tissues. Regional magnification images were shown below. **g** Immunofluorescence staining of B4GALNT1 and CD4-positive cells in B4GALNT1^high^ and B4GALNT1^low^ tissues. **h** Correlations analysis on B4GALNT1 levels with the numbers of CD163 + macrophages and CD4 + T cells. **i** CellChat analysis for the cross-talk between different cell types. The cross-talk strengths determined by interaction weight were determined among B4GALNT1-positive malignant cell, B4GALNT1-negative malignant cell, TAMs, T cells and other cell types in GSE125449 (Up left) and GSE149614 (Up right). And the CellChat differences between B4GALNT1-positive and B4GALNT1-negative malignant cells were also analyzed (down). Data are represented as the mean ± SD (*n* = 6), Statistical analysis was performed using Student’s t-test

We subsequently explored the precise mechanisms in B4GALNT1 regulating tumor immunity. Since B4GALNT1 levels are significantly associated not only with the levels of human leukocyte antigen (*HLA)* class *I* antigens but also with those of *HLA* class *II* members, which are preferentially expressed on various antigen-presenting cells (APCs) (Fig. S2), we speculated that tumor cell-derived B4GALNT1 may be involved in the recruitment or infiltration of some immune cells, especially APCs. Based on the caner-immunity cycle system [22, 23], we analyzed the activation differences of tumor immune steps between the B4GALNT1^high^ and B4GALNT1^low^ groups, and determined the highly activated pathways in B4GALNT1^high^ tumors as the recruitment of immunosuppressive cells, such as Regulatory T (Treg) cells, T helper 2 (Th2) cells, tumor-associated macrophages (TAMs), and myeloid-derived suppressor cells (MDSCs) (Fig. 5a). Multiple tools, including XCELL, TIMER, QUANTISEQ, MCPcounter, EPIC and CIBERSORT, were further utilized to evaluate the infiltration of these immunosuppressive cells in the HCC microenvironment, and the subsequential correlation analysis confirmed that some immunosuppressive subpopulations, such as M2-type TAMs and Th2 cells, were positively correlated with B4GALNT1 levels, whereas negative correlations were observed in CD8 + naive T cells that confer tumor-killing potentials (Xcell, *R* = −0.240, *p* < 0.001) (Fig. 5b). Further transwell assays supported that overexpressing B4GALNT1 in HCC tumor cells significantly promoted the infiltration of THP-1-derived human macrophages (*p* < 0.001) (Fig. 5c). Similar results were also observed when tumor cell-derived culture media was used on macrophages (*p* < 0.001) and Peripheral Blood Mononuclear Cells (PBMC)-derived human CD4 + T cells (*p* = 0.0025) (Fig. 5d and e). Moreover, IHC staining of HCC tumor tissues from our cohort also revealed significant correlations of B4GALNT1 levels with the numbers of infiltrating CD163 + TAMs (R^2^ = 0.06, *p* = 0.019) and CD4 + T cells (R^2^ = 0.05, *p* = 0.038) (Fig. 5f, g and h). CellChat analysis on single-cell data returned similar results, that B4GALNT1-positive malignant cells conferred stronger interaction strengths with other cell types, especially with TAMs and T cells (Fig. 5i and Fig. S3). Taken together, these data demonstrate the roles of B4GALNT1 in immunoregulation and indicate that B4GALNT1 promotes the infiltration of TAMs and CD4 + T cells via modulating intercellular communication.

### B4GALNT1 promotes immunosuppression by increasing secreted phosphoprotein 1 (SPP1)

**Fig. 6:**
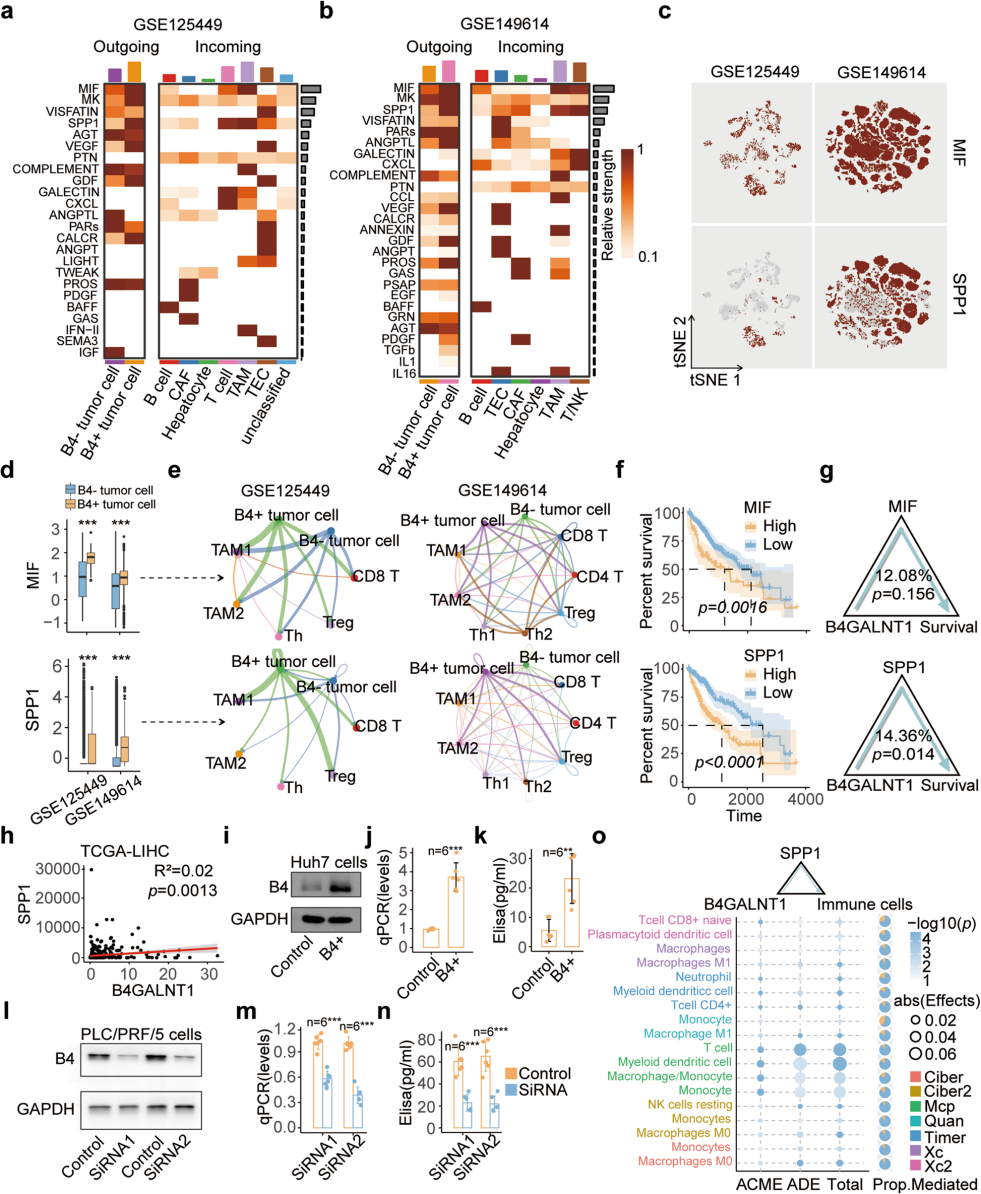
Crucial roles of SPP1 in B4GALNT1-associated immunosuppression. **a**-**b** Key outgoing and incoming communication patterns and molecules responding the upregulation of B4GALNT1 in malignant cells from GSE125449 and GSE149614 were determined by CellChat analysis. **c** The expression patterns of MIF and SPP1 in different cell types from two single-cell datasets. **d** Expressional differences of MIF and SPP1 between B4GALNT1-positive (B4+) and B4GALNT1-negative (B4-) malignant cells. **e** The cross-talk strength of MIF and SPP1 signals between malignant cells and immune cell subgroups. **f** Kaplan-Meier analysis on the overall survival of HCC patients based on the expression of MIF or SPP1 in TCGA-LIHC. **g** The mediation analysis on SPP1 and MIF between B4GALNT1 expression and overall survival. **h** Expressional correlation between SPP1 and B4GALNT1 in TCGA-LIHC. **i** Overexpression of B4GALNT1 in Huh7 cells determined by WB analysis. **j**-**k** Elevation on the synthesis (**j**) and secretion (**k**) of SPP1 in B4GALNT1-overexpressed Huh7 cells determined by qPCR and ELISA. **l** B4GALNT1-knockdown in PLC/PRF/5 cells determined by WB analysis. **m-n** Expressional changes of SPP1 at mRNA (**m**) and protein (**n**) levels in B4GALNT1-knockdown cells determined by qPCR and ELISA. **o** Mediation analysis on the mediating effects between B4GALNT1 expression and the recruitments of different immune cells in TCGA-LIHC. Data are represented as the mean ± SD (*n* = 6), Statistical analysis was performed using Student’s t-test, **p < 0.01, ***p < 0.001

To investigate how B4GALNT1 regulated the infiltration of immune cells, we proceed the CellChat analysis, and found that the outgoing signals of macrophage migration inhibitory factor (MIF) and SPP1 were significantly increased in B4GALNT1-positive malignant cells, and the major incoming cell types with activated MIF and SPP1 pathways were TAMs and T cells (Fig. 6a, b and Fig. S4), indicating MIF and SPP1 as the major secreting molecules during B4GALNT1-involved immunoregulation. Both MIF and SPP1 are pleiotropic proteins. MIF is involved in leukocyte recruitment, inflammation, immune responses, cell proliferation, tumorigenesis, and counter-regulation by glucocorticoids [24], and SPP1 has cytokine, chemokine, and signal transduction functions. Both of these proteins have been reported to regulate immunosuppression [25, 26]. Single-cell data confirmed that MIF and SPP1 were distributed mainly in malignant cells (Fig. 6c), and their levels were significantly greater in B4GALNT1-positive malignant cells than in negative cells (Fig. 6d). Deep subclustering divided TAMs and T cells into several subsets, including TAM1, TAM2, T helper 1 (Th1), Th2, and Treg cells (Fig. S5), and CellChat analysis on these subsets revealed that, similar to the results for major cell types, B4GALNT1-positive malignant cells still conferred stronger interactions with TAM and T-cell subpopulations compared with B4GALNT1-negative ones (Fig. S6). And among all the SPP1-receiving T-cell subsets, immunosuppressive Treg and Th2 cells elicited greater responses than what Th1 cells did (Fig. 6e and Fig. S7). Intriguingly, although the upregulation of MIF (*p* = 0.0016) and SPP1 (*p* < 0.001) was significantly associated with worse overall survival in the TCGA-LIHC cohort (Fig. 6f), the changes in SPP1 signals between malignant cells and immune subsets were greater than those in MIF signals (Fig. S7), and mediation analysis revealed that the prognostic effects of B4GALNT1 were mediated only by SPP1 (mediated proportion = 14.36%, *p.ACME* = 0.014) instead of MIF (mediated proportion = 12.08%, *p.ACME* = 0.156) (Fig. 6g). To verify the effects of B4GALNT1 on SPP1, sequencing data of 424 HCC patients from TCGA-LIHC cohort were analyzed, and the positive correlation between the levels of B4GALNT1 and SPP1 was also observed (Fig. 6h). Furthermore, overexpressing B4GALNT1 in Huh7 cells significantly elevated the synthesis and secretion of SPP1 (Fig. 6i, j and k). And B4GALNT1 knockdown in PLC/PRF/5 cell line, that conferred relatively higher levels of B4GALNT1 than other cell lines, significantly decreased the expression of MIF and SPP1 at both mRNA and protein levels (Fig. 6l, m, n and Fig. S8a, b). The mediation analysis on SPP1 in B4GALNT1 regulating immune cell compositions confirmed that SPP1 significantly mediated the recruiting effects of B4GALNT1 on TAMs and CD4 + T cells (Fig. 6o). These data indicate that B4GALNT1 in malignant cells remodels the immunosuppressive microenvironment by increasing SPP1 signaling (Fig. S9).

### B4GALNT1 regulates SPP1 levels by enhancing Hes family BHLH transcription factor 4 (HES4) activity

**Fig. 7:**
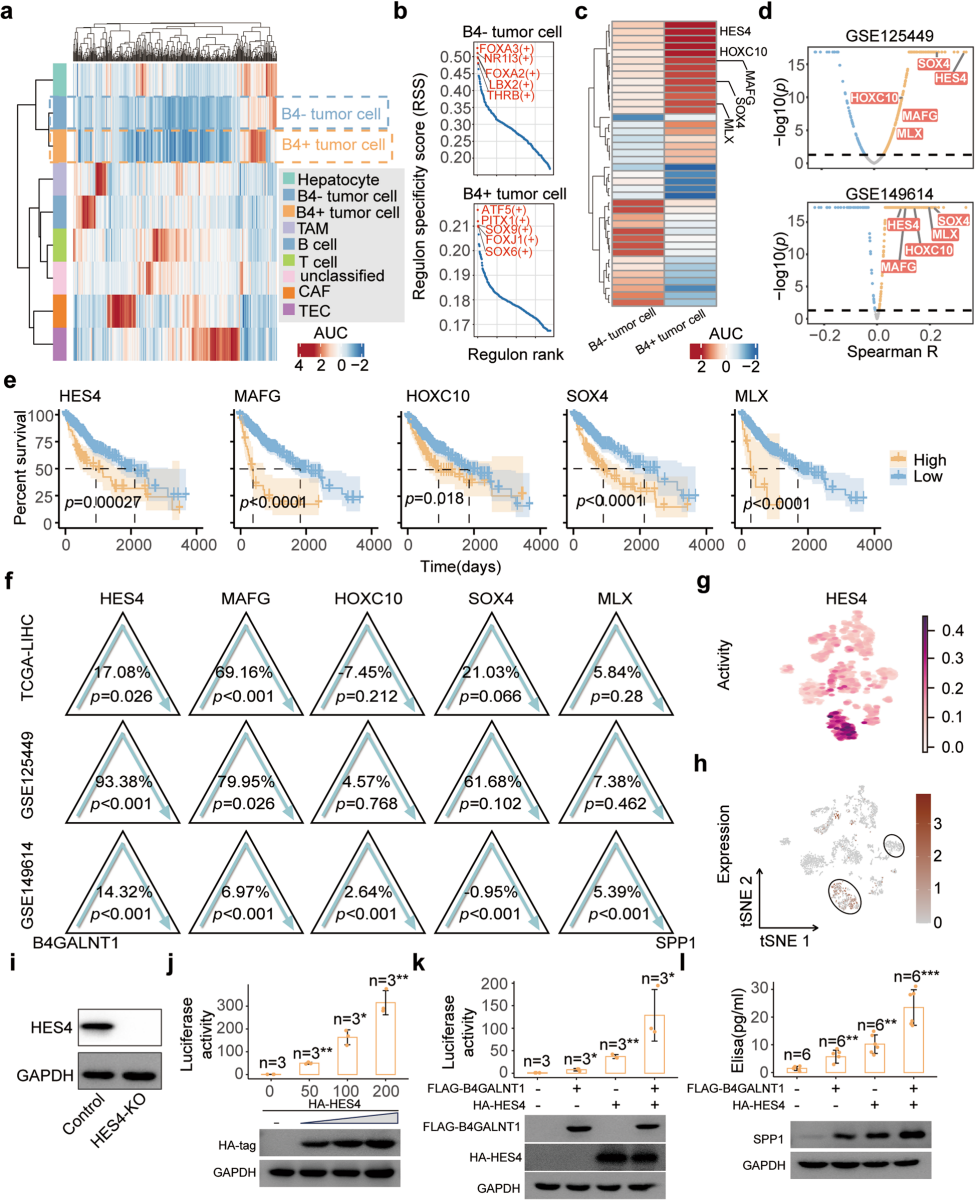
Determination of HES4 as the key TF responsible for the elevation of SPP1 by B4GALNT1. **a** Scenic analysis on different cell types in GSE125449. **b** The top 5 TFs with highest regulon specificity score (RSS) in B4GALNT1-positive or -negative malignant cells. **c** Activation differences of the TFs between B4GALNT1-positive and -negative malignant cells. **d** Correlation analysis on the candidate regulons targeting SPP1 in the malignant cells from GSE125449 and GSE149614. **e** Kaplan-Meier analysis on the overall survival of HCC patients in TCGA-LIHC cohort based on the expression of HES4, MAFG, HOXC10, SOX4 and MLX. Log-rank test was used. **f** Mediation analysis on the candidate TFs between B4GALNT1 and SPP1 were performed in TCGA-LIHC, GSE125449 and GSE149614 datasets. **g**-**h** Activating (**g**) and expressional (**h**) distribution of HES4 in different cells types from GSE125449. **i** Knockout efficiency targeting HES4 in PLC/RCF/5 cells determined by WB analysis. **j-k** Luciferase analysis on the transcriptional activity of SPP1 in HES4-KO cells under the transfection of increased amount of HES4-expression plasmid (*n* = 3) (**j**) or co-transfection of HES4 and B4GALNT1 (*n* = 3) (**k**). **l** The protein levels of SPP1 under the upregulation of HES4 and/or B4GALNT1 were determined by WB analysis (down) and ELISA (*n* = 6) (up). Data are represented as the mean ± SD, Statistical analysis was performed using Student’s t-test, *p < 0.05, **p < 0.01, ***p < 0.001

To further investigate how B4GALNT1 regulates the expression of SPP1, SCENIC modulator inference analysis was performed to identify the transcriptional regulators specifically responding to the upregulation of B4GALNT1 (Fig. 7a and b). Although Upstream Transcription Factor 2, C-Fos Interacting (USF2) and Retinoid X Receptor Gamma (RXRG) were determined as the key Transcription Factors (TFs) responsible for the transcriptional changes of SPP1 according to the RcisTarget reference in SCENIC analysis [27], only slight changes in the activity of USF2 and RXRG were observed under the upregulation of B4GALNT1 (Fig. S10). Thus, we expanded our screening criteria to all other TFs in the RcisTarget reference, and identified 139 activated TFs responding to B4GALNT1 upregulation (Table S4 and Fig. 7c). At expression levels, most of these TFs were positively correlated with B4GALNT1 in malignant cells, and some of them were screened out correlated with SPP1 in both single-cell datasets and significantly associated with worse overall survival in the TCGA-LIHC cohort (Fig. 7d and e). Among these candidates, HES4 and MAFG significantly mediated the effects of B4GALNT1 on SPP1 in all datasets (Fig. 7f). In single-cell datasets, the mediated proportions of HES4 were greater than those of MAFG, indicating that in malignant cells, B4GALNT1 elevates SPP1 levels mainly through the activation of HES4 rather than MAFG. However, in the bulk-seq data of tumor tissues from TCGA-LIHC, MAFG (69.16%) had a greater mediating effect than HES4 (17.08%), possibly because of the higher distribution of MAFG in nonmalignant cells. And the activation and expression distributions of HES4 and MAFG (Fig. 7g and h) verified that the distribution proportions of HES4 in malignant cells were greater than those of MAFG (Fig. S11a, b and S12). Therefore, we focused on HES4 as the key TF in B4GALNT1 regulating SPP1 expression. To verify the effects of HES4, luciferase assays were performed in HES4 Knockout (HES4-KO) tumor cells (Fig. 7i). With increasing doses of HES4-expressing plasmids, the transcriptional activity of SPP1 significantly increased (Fig. 7j). Importantly, the overexpression of B4GALNT1 alone in the absence of HES4 resulted in only a small increase in luciferase activity, whereas the co-transfection of B4GALNT1 with HES4 dramatically increased activity (Fig. 7k). Similar results were observed in detecting protein and secreted levels of SPP1 (Fig. 7l), demonstrating that B4GALNT1 could promote the synthesis and secretion of SPP1 from tumor cells via HES4.

### B4GALNT1 enhances HES4 activity by regulating the phosphorylation of the Mitogen-Activated Protein Kinase (MAPK) signaling pathway

**Fig. 8:**
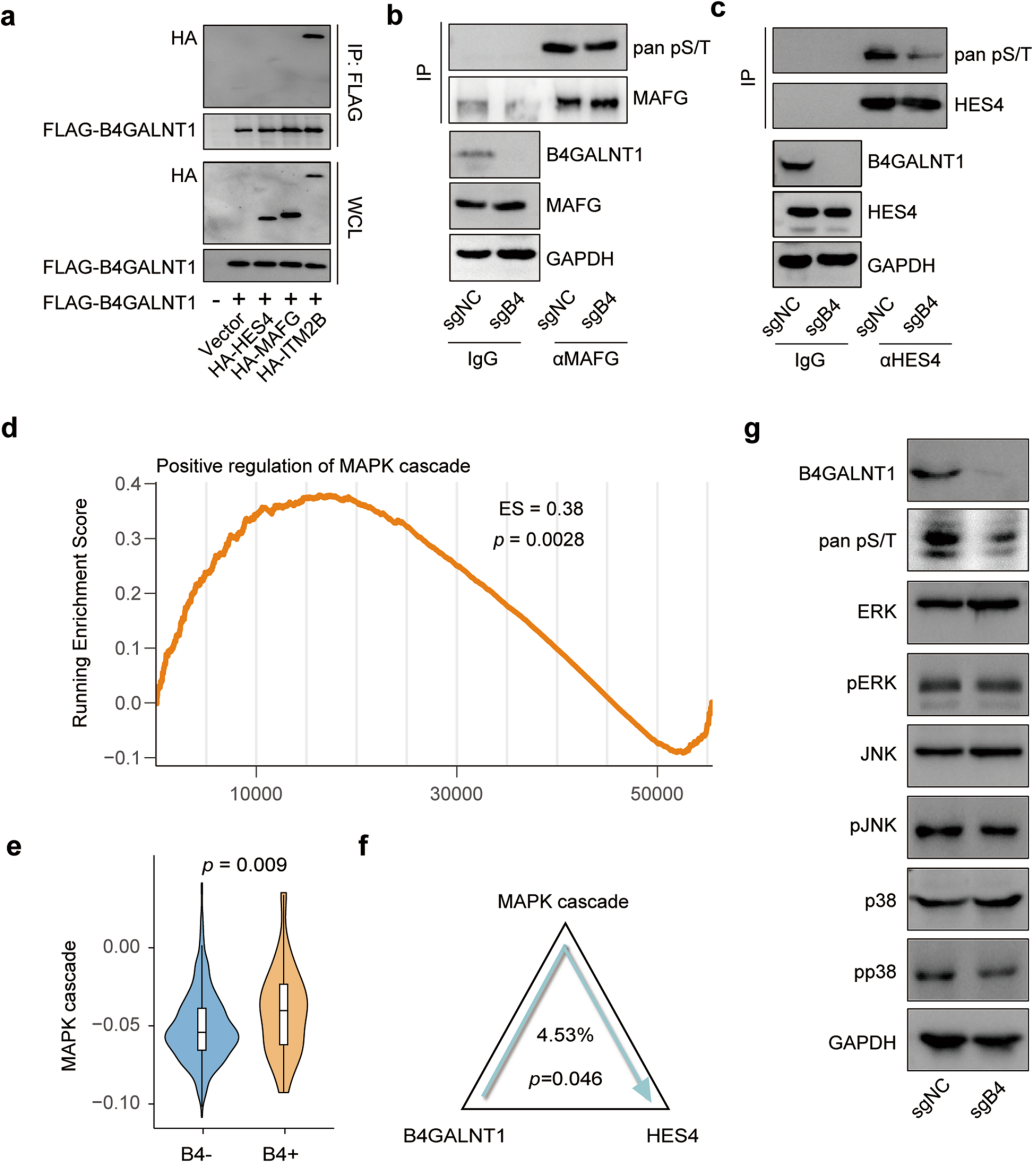
B4GALNT1 regulates HES4 phosphorylation and activity via the MAPK signaling pathway. **a** Coimmunoprecipitation (co-IP) assays to assess the physical interaction between B4GALNT1 and HES4 or MAFG. **b**-**c** Phosphorylation-specific IP assays showed that B4GALNT1 knockdown resulted in changes in HES4 and MAFG phosphorylation levels. **d** Gene set enrichment analysis (GSEA) of B4GALNT1 overexpression correlating with the upregulation of the MAPK signaling pathway. **e** Comparison of MAPK pathway between B4GALNT1 positive and B4GALNT1 negative malignant tumor cells in GSE125449. **f** Mediation analysis on the MAPK pathway between B4GALNT1 expression and the activity of HES4 were performed in GSE125449. **g** Western blot of MAPK pathway components showed changes in the phosphorylation of JNK, p38 and ERK in B4GALNT1 knockdown cells

To elucidate the mechanism by which B4GALNT1 enhances HES4 activity, we first performed coimmunoprecipitation (co-IP) assays to determine the direct physical interaction between B4GALNT1 and HES4 but failed to observe positive results, indicating that B4GALNT1 could not directly regulate the activity of HES4 (Fig. 8a). Since HES4 can be phosphorylated [28] and the phosphorylation of another HES family member, HES1, has been reported to change its transcriptional activity [29], we next conducted phosphorylation-specific Immunoprecipitation (IP) assays on HES4, and found that in B4GALNT1 knockout (B4-KO) cells, the overall phosphorylation level of HES4 was notably lower than that in control cells (Fig. 8b and c), indicating the indirect effects of B4GALNT1 on the phosphorylation and activity of HES4. Considering that MAFG was another major transcription factor in the B4GALNT1-SPP1 axis (Fig. 7) and that the phosphorylation could also enhance the activity of MAFG [30], we also performed IP analysis on MAFG. Similarly, no direct interaction was observed between B4GALNT1 and MAFG, and indirect phosphorylation of MAFG could be regulated by B4GALNT1 (Fig. 8a, b and c). Intriguingly, despite the similar change patterns, the phosphorylation change in HES4 was more pronounced than that in MAFG under B4GALNT1 silencing, which is consistent with previous conclusions on the downstream transcriptional majority of B4GALNT1 as HES4 instead of MAFG.

We further investigated the signaling pathways through which B4GALNT1 regulates the phosphorylation of HES4. Considering the reported downstream MAPK signals of B4GALNT1 in promoting tumor progression [31], we hypothesized that the MAPK signaling pathways may also be involved in B4GALNT1-HES4 regulation. The GSEA results revealed that the upregulation of B4GALNT1 was associated with positive regulation of the MAPK cascade, including the JNK and extracellular regulated protein kinases (ERK) signals (Fig. 8d and Fig. S13). The GSVA results also showed that B4GALNT1-positive malignant tumor cells exhibited higher MAPK signaling pathway activity compared to B4GALNT1-negative cells (Fig. 8e). Mediation analysis further demonstrated that the MAPK signaling pathway mediated the regulation from B4GALNT1 to the activity of HES4 (Fig. 8f). Moreover, in vitro data proved significant reductions in the phosphorylation levels of several key signal transductors in the MAPK pathway, including JNK, p38, and ERK, under B4GALNT1 silencing (Fig. 8g). These findings suggest that B4GALNT1-mediated regulation of the MAPK pathway, which involves both p38/JNK and ERK signaling, might be responsible for the change in HES4 phosphorylation, eventually modulating the transcriptional activity of HES4.

### Targeting B4GALNT1 enhances the tumor-killing efficiency of the PD-1-targeting strategy

**Fig. 9:**
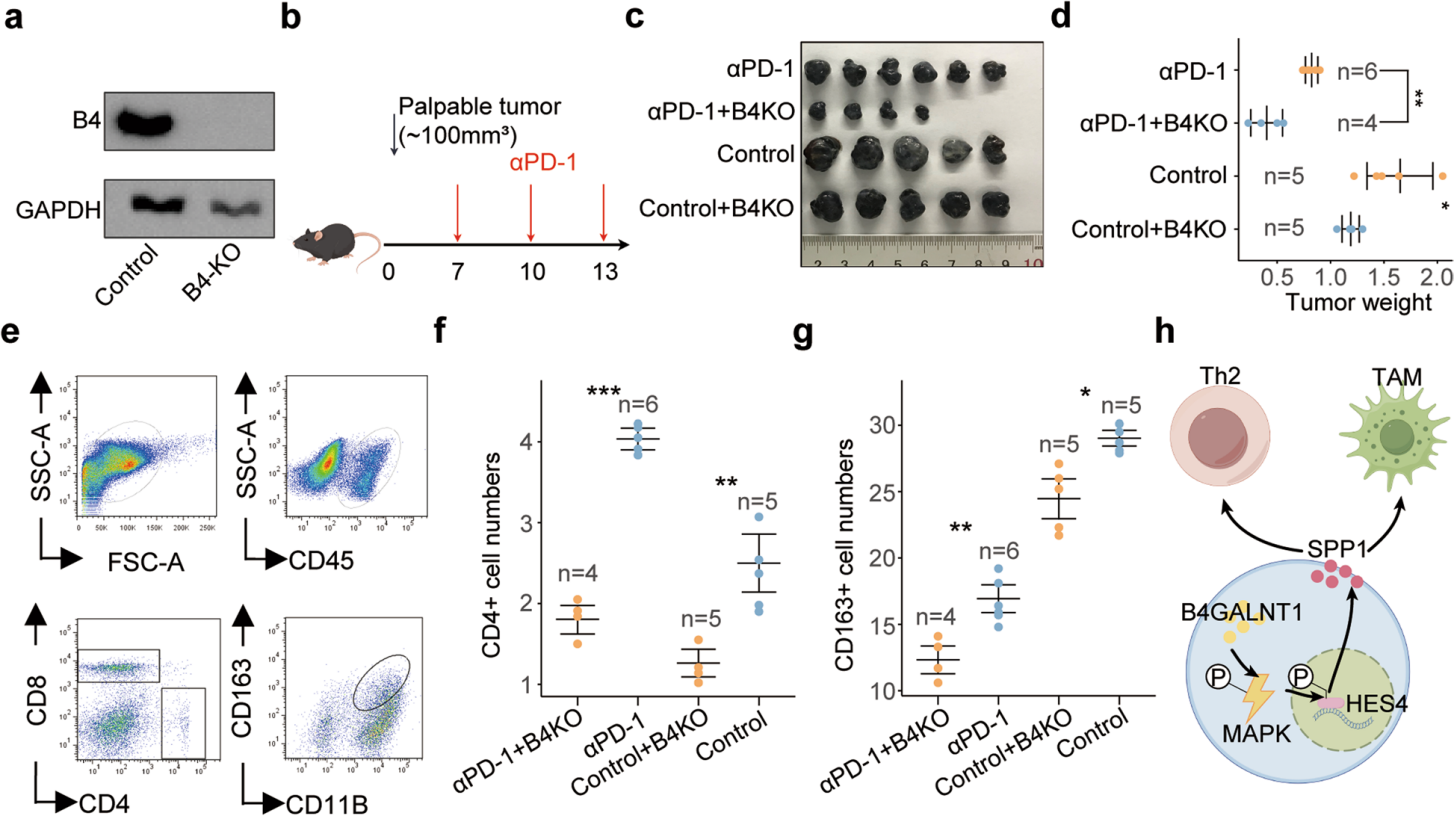
In vivo evaluation on the effects of B4GALNT1-depletion on tumor growth and immunotherapy. **a** Knockout efficiency targeting B4GALNT1 (B4) in Hepa1-6 cells determined by WB analysis. **b** The flow chart of the in vivo experiments. **c** Tumor weight of mice under different treatments (αPD-1 (*n* = 6), αPD-1 + B4GALNT1^KO^ (*n* = 4), control (*n* = 5), control + B4GALNT1^KO^ (*n* = 5)). **d** Statistic analysis on the tumor weights under different treatments. **e** Representative images of flow cytometry analysis on CD4^+^CD8^−^CD45^+^ T cells and CD163^+^CD11B^+^CD45^+^ macrophages in the xenografts. **f**-**g** Statistical analysis for the numbers of CD4 + T cells (**f**) and CD163+ (**g**) macrophages in the xenografts under different treatments (αPD-1 (*n* = 6), αPD-1 + B4GALNT1^KO^ (*n* = 4), control (*n* = 5), control + B4GALNT1^KO^ (*n* = 5)). **h** The schematic diagram for the regulation of B4GALNT1-HES4-SPP1-TAM/Th2 axis (By Figdraw). Data are represented as the mean ± SD, Statistical analysis was performed using Student’s t-test, *p < 0.05, **p < 0.01, ***p < 0.001

To further verify the effects of *B4GALNT1* on tumor progression and immune regulation, we generated a B4GALNT1-KO cell line and tested its tumor growth in a mouse model (Fig. 9a and b). Our results confirmed that B4GALNT1 depletion significantly reduced tumor weight (*p* < 0.05, Fig. 9c and d), and the absence of B4GALNT1 led to a reduction in CD4 + T cells and CD163 + TAMs (Fig. 9e, f and g). Intriguingly, we also observed decreased CD8 + T cells in B4GALNT1-KO xenografts (Fig. S14), indicating that the upregulation of B4GALNT1 increases the total number of CD8 + T cells. Considering the immunosuppressive effects of B4GALNT1 described above, we speculated that despite the increase in total CD8 + T-cell numbers, B4GALNT1 may exert additional suppressive effects on CD8 + T cells via immune checkpoints. Consistent with our speculation, TCGA-LIHC data revealed a significant correlation between B4GALNT1 and PD-1 (Fig. S15), and CellChat analysis revealed that B4GALNT1-positive malignant cells had a stronger outgoing interaction with PD-1-positive CD8 + T cells than with PD-1-negative CD8 + T cells (Fig. S16), supporting the role of B4GALNT1 in regulating checkpoints. Therefore, we next evaluated the effects of targeting B4GALNT1 on the tumor-killing efficiency of the PD-1-targeting strategy. In xenograft-bearing mice treated with antibodies against PD-1, we observed significant enhancement of B4GALNT1 depletion on the tumor-killing effects of anti-PD-1 antibodies (Fig. 9b), indicating that targeting B4GALNT1 may be a valuable auxiliary approach for modifying current immunotherapy (Fig. 9h).

## Discussion

HCC is the sixth most prevalent cancer and the third leading cause of cancer-related death, which emphasizes the importance of an accurate prognosis before HCC treatment [32]. However, owing to its underlying cirrhosis and marginal utility [33], HCC prognosis is so complicated that traditional HCC staging systems, such as the TNM system, Child‒Pugh classification, Okuda staging and Barcelona–Clinic Liver Cancer (BCLC) staging classification, remain inefficient [34, 35]. Therefore, the development of a more effective prognostic system, especially a system involving specific molecular markers associated with tumor progression, is urgently needed for HCC patients. GD2 [9, 10] and GM2 [11, 36, 37] have been reported to be markers of HCC and are associated with HCC prognosis. Since they share a common synthase B4GALNT1, using B4GALNT1 may be more sensitive for prognosis than using those downstream gangliosides. Actually, there has been one study revealed the association between B4GALNT1 levels and the survival in patients from TCGA-LIHC cohort via KM analysis [21]. But given the predominantly European ancestry of the populations in TCGA cohort, and considering the genetic background differences across ethnicities, it is currently uncertain whether B4GALNT1 is also associated with HCC prognosis in Asian populations. Here, by analyzing the Chinese liver cancer population, our study confirms the prognostic value of B4GALNT1 across different ethnicities for the first time. Moreover, in addition to the survival analysis, we integrated B4GALNT1 levels with the conventional TNM staging system in ROC analysis and nomogram modeling, and demonstrated that the inclusion of B4GALNT1 significantly improved the predictive accuracy of the TNM classification on prognosis. These findings verify the clinical value of B4GALNT1 as an independent prognostic biomarker, offer valuable insights for clinical decision-making, and supplement extra quantitative tool for personalized risk assessment.

Although most gangliosides, including GD2 and GM2, in the serum are secreted from the liver, their pathological functions in the liver are poorly understood, especially their roles in HCC. It has only been reported that increased GD2 can be detected in the plasma of patients with HCC [38]. In accordance with this report, our study demonstrated that the key GD2/GM2 synthase B4GALNT1 is upregulated in HCC, which would result in increases in GD2 and GM2. Moreover, our data also revealed an association between B4GALNT1 expression and overall survival in HCC patients, suggesting that the ganglioside profiles altered by aberrant B4GALNT1 expression are critical for HCC progression.

As one of the key products of B4GALNT1, GD2 is closely associated with both the adhesive and antiadhesive properties of cells. GD2 could interact with the extracellular matrix protein tenascin-C in cancerous tissues to disrupt cell adhesion and inhibit focal adhesion formation [39], thus elevating the metastatic potential of tumor cells. And such promoting effects of GD2 on the motility of tumor cells have been verified in vitro [40]. Meanwhile, GD2 is involved in the complex with focal adhesion kinase (FAK) [41], which has been reported to promote migration, angiogenesis [42], anchorage-independent growth, and tumor cell survival during metastasis [43, 44]. In addition to these tumor-cell-centric functions, GD2 also plays roles in regulating the tumor immune microenvironment. GD2 was reported to inhibit T-cell reactivity both in vitro and in vivo [45, 46]. And tumor-derived GD2 suppresses T-cell proliferation [46], which is beneficial for the immune evasion of tumor cells. Although this study did not directly investigate the immune effects of GD2, the upregulation of B4GALNT1 in tumor cells observed in our results could lead to an increase in GD2 levels. Given that GD2 has been reported to influence downstream phosphorylation [47], and the effects of B4GALNT1 on SPP1 depend on the phosphorylation signal transductions from B4GALNT1 to HES4 as determined in this study, the impacts of B4GALNT1 on SPP1 secretion and subsequent immune environmental alternations were likely mediated by GD2. These findings suggest a potential role of GD2 in the immune dysregulation associated with B4GALNT1 upregulation in HCC, and the underlying mechanisms require further investigation in future studies.

To investigate the detailed roles of B4GALNT1 in altering HCC tumor microenvironment (TME), we performed bioinformatic analysis, and found that B4GALNT1 expression was positively correlated with the numbers of TAMs and Th2 cells in TME. Liver macrophages are involved in the development and progression of several liver diseases including HCC, and act as tolerizing antigen-presenting cells to maintain or resolve inflammation by producing various cytokines, chemokines, and mediators [48]. Liver macrophages are widely known to be tumorigenic, suppress antitumor immunity and favor tumor establishment [49–51]. The major subpopulations of liver macrophages are associated with poor survival in HCC patients [52, 53]. In addition to macrophages, Th2 cells are also well known to mediate immunosuppression [54], and reportedly abolish the antitumor response when Th2 cells dominate the TME [55]. Therefore, increased numbers of macrophages and Th2 cells resulting from the upregulation of B4GALNT1 reshape the TME into an immunosuppressive environment, which ultimately promotes tumor progression.

During TME reshaping, some key cytokines secreted from tumor cells drive the compositional alteration of immune cells [51]. Here, we identified SPP1 as the critical secretion factor for the process by which tumor B4GALNT1 modulates the recruitment or polarization of macrophages and Th2 cells. Aberrant upregulation of SPP1 has been observed in different solid tumors such as lung cancer [56], glioblastoma [57], breast cancer [58], and pancreatic cancer [59]. SPP1 functions in tumor-associated inflammation and can promote tumor progression [60]. SPP1 regulates host immunity during the chronic inflammation associated with cancer development [61] and is capable of promoting proliferation and preventing the apoptosis of tumor cells [62]. In HCC, SPP1 signals can crosstalk with the colony-stimulating factor-1 (CSF1) pathway to mediate the TME switch from Th1 to Th2, which is vital for tumor progression [25]. In SPP1-KO tumors, both Th2-derived cytokines and markers associated with M2 macrophages were reduced [25]. SPP1 can upregulate PD-L1 to mediate M2 polarization of macrophages and promote immune escape in lung adenocarcinoma [63]. SPP1 was also reported to promote the infiltration of TAMs in glioma [64], which is similar to our results in B4GALNT1-overexpressing HCC cell lines. Therefore, SPP1 is critical for the effects of B4GALNT1 on the polarization and recruitment of M2 macrophages and Th2 cells, which eventually leads to immune escape and tumor progression in HCC.

To determine how B4GALNT1 elevates the expression of SPP1, we investigated which downstream TFs of B4GALNT1 are responsible for the increase in SPP1, and HES4 was determined to be the key TF. HES4 is a HES family BHLH transcription factor. Previous studies have shown that increased HES4 expression is associated with poor prognosis in acute‒chronic liver failure (ACLF) [65] and osteosarcoma (OS) patients [66]. However, the role of HES4 in HCC progression remains unclear. This study revealed that HES4 is involved in the immunosuppression of HCC by promoting the transcriptional activity of SPP1 and that its own activity can be promoted by B4GALNT1 in malignant cells. Our data also revealed that, in HCC patients, high levels of HES4 are associated with reduced overall survival. Therefore, our data identified HES4 as a novel tumor-promoting TF in HCC.

On the basis of our discovery of the B4GALNT1-HES4-SPP1-TAM/Th2 axis and considering the reported effects of SPP1 [62, 63], TAM [67] and Th2 [68] on immune escape, we also speculated the potential benefit of targeting this axis for HCC immunotherapy. Since the efficacy of immune therapy depends on the tumor immune microenvironment, tracing the origin responsible for the alteration in the immune environment is necessary to select appropriate inhibitors [69]. In this study, B4GALNT1 was identified as an original factor leading to the immunosuppressive microenvironment in HCC, and could be treated as a target. In addition, our data revealed a correlation between B4GALNT1 and PD-1 levels in tumor tissues, and strong crosstalk between B4GALNT1-positive malignant cells and PD-1-positive CD8 + T cells. Considering that PD-1 itself plays an important role in immunotherapy, regulating inhibitory pathways through ligand‒receptor interactions to maintain immune tolerance and modulate physiological responses [70, 71], these results suggest that targeting B4GALNT1 could improve PD-1-targeting immunotherapy in different ways. While PD-1 inhibitors have indeed marked a milestone in cancer treatment, resistance to these therapies remains a challenge for some patients [72]. Some individuals may exhibit primary resistance to PD-1 inhibitors, possibly due to a lack of tumor immunogenicity or the influence of immunosuppressive elements within the tumor microenvironment [73]. Compared with monotherapy, combination targeted therapies typically yield better outcomes [74]. Combination therapies can target suppressive cells or molecules in the tumor microenvironment, increasing the efficacy of PD-1 inhibitors and enhancing immune system antitumor responses [75, 76]. Here, our in vivo data demonstrated that silencing B4GALNT1 significantly enhances the tumor-killing efficiency of the PD-1 targeting strategy, indicating that B4GALNT1 is a strong candidate for combined immune therapy approaches. This combined strategy could have greater potential than monotherapy does, and future clinical studies should further investigate the efficacy of this approach and its effects on patient outcomes. This study offers innovative opportunities for improving HCC immunotherapy, and provides theoretical evidence for advancing personalized and precise therapeutic strategies to ultimately improve patient outcomes.

In conclusion, this study revealed the upregulation of B4GALNT1 in HCC tumor cells, which could be used as a valuable prognostic predictor, and revealed the functions of B4GALNT1 in microenvironment remodeling in HCC via the HES4-SPP1-TAM/Th2 axis. We also demonstrated the benefit of B4GALNT1 depletion in PD-1-targeted immunotherapy, which enhances the understanding of HCC progression and provides a novel auxiliary strategy for HCC immunotherapy.

## Materials and methods

### HCC patient samples

Approval for the use of human tissue and clinical data was granted by the ethics committee of The First Affiliated Hospital of Wannan Medical College (Wuhu, China), with all patients providing written informed consent per the Helsinki Declaration. Ninety-three HCC patients without prior therapy were recruited between 2010 and 2012. Follow-up ended on December 9, 2016, with patients monitored every three months and a median follow-up of 33.3 months (range: 0.8 to 60.4 months). Of the samples, 21 were selected for Western blot and qPCR analyses.

### Data source

RNA sequencing data of TCGA (https://portal.gdc.cancer.gov) for HCC were retrieved from the Xena Toil RNA-Seq Recompute Compendium (https://toil.xenahubs.net). Additionally, bulk-seq transcriptome data (GSE20140 [77], GSE22058 [78], GSE25097 [79], GSE36376 [80], GSE54236 [81] and GSE76427 [82]) and single-cell transcriptome data (GSE125449 [83] and GSE149614 [84]) were retrieved from the Gene Expression Omnibus (GEO) database (https://www.ncbi.nlm.nih.gov/geo/).

### In vivo experiments

Mice were inoculated subcutaneously with 0.5–1 × 10^6^ Hepa1-6 control cells or B4GALNT1-KO cells in 100 µl. Anti-mouse-PD-1 or rat Immunoglobulin G2a (IgG2a) isotype mAbs (Bioxcell, USA) were given intraperitoneally at a dose of 150 µg per mouse on day 7 after tumor cell inoculation, then every 3 days for the duration of the experiments. All animal procedures were approved by the University of Fudan Committee on Use and Care of Animals. Tumor size did not exceed 2 cm in any dimension in any experiment.

### Generation of knockout cell lines

Knockout (KO) cell lines were generated as previously reported [85]. Briefly, sgRNAs were designed in http://cistrome.org/crispr-focus/, and sgRNAs targeting B4GALNT1 were inserted into a two-vector lentiviral system (lentiCRISPR-V2). After lentivirus production in 293T cells, collected lentivirus were used for tumor cell infection and pooled generation under the treatment of 10 µg/ml blasticidin (Yeasen Biotech, China). Tumor cells were infected for 2 days, followed by selection for 4 days. KO efficiency was confirmed via Western blotting.

sgRNA Oligo sequence:

- Mouse sgRNA1: CTAGCGCATAGAGGGCCCGG
- Mouse sgRNA2: CAGGAGACCCAGCGAGGCGC
- Mouse sgRNA3: TGAACCTCCACACCCTGCAG
- Mouse sgCtrl: GACTGTGGCGTAGTTCCCGG
- Human HES4 sgRNA1: GTTCTGGGCAAGTACCGCGC
- Human HES4 sgRNA2: CCAAGCCGGTCATGGAGAAG
- Human HES4 sgRNA3: CCTTCTCCAGCTTCGAGTGG
- Human sgCtrl: GGCGGTCCTTACAAGACGGG

### Statistical analysis

All analysis was performed with R software (Ver. 4.2.0). Results were presented as Means ± standard deviation with at least three replicates for each sample. Optimal cut-off value of B4GALNT1 expression was determined by ROC curve analysis. Pearson’s Chi-square test was used to identify the correlation between B4GALNT1 expression and other clinicopathological factors. Nomogram was constructed using the “rms” package. According to the risk points in the nomogram, we stratified the patients into three groups to verify the prognostic efficiency. The low-risk group included patients with lower total scores, accounting for 25% of all patients; the medium-risk group included patients with medium total scores, accounting for 50% of all patients; and the high-risk group included patients with higher total scores, accounting for 25% of all patients. Survival probability was determined by KM curve and the differences between groups were assessed by Log-rank test. Univariate and multivariate survival analysis were applied using Cox regression. To examine the performance of the nomogram, calibration plot was generated. Akaike information criterion (AIC) and Harrell’s concordance index (C-index) were used to assessing the predictive value of the parameters. Differences between groups were determined with Student’s t test. Statistical significance was set at two-tails *p* < 0.05.

See Supplementary Methods for more information on methods.

## Supplementary Information

Supplementary Material 1.

Supplementary Material 2.

Supplementary Material 3.

Supplementary Material 4.

Supplementary Material 5.

## Data Availability

The data used to support the results of the present study are available from the corresponding author upon reasonable request.
