# Supplementary material for: Glycosyltransferase B4GALNT1 promotes immunosuppression in hepatocellular carcinoma via the HES4-SPP1-TAM/Th2 axis
Source: Mol Biomed. 2024 Dec 1;5:65. doi: 10.1186/s43556-024-00231-w (PMC11608210; doi:10.1186/s43556-024-00231-w)

**Supplementary materials**

**Glycosyltransferase B4GALNT1 promotes immunosuppression in hepatocellular carcinoma via the HES4-SPP1-TAM/Th2 axis**

Zhifeng Wang^1,3,4,*^, Jiaxin Liu^1,4,*^, Xiaoming Wang^3,*^, Qingyun Wu^1,4^, Qiao Peng^5^, Tianxiao Yang^1^, Xuehui Sun^1,4^, Xiaofeng Wang^1,4^, Yilin Wang^2,§^, Weicheng Wu^1,4,§^

**Authors’ Affiliations**

^1.^ Human Phenome Institute, and State Key Laboratory of Genetic Engineering and MOE Key Laboratory of Contemporary Anthropology, School of Life Sciences, Fudan University, Shanghai, China.

^2.^ Department of Hepatic Surgery, Fudan University Shanghai Cancer Center; Department of Oncology, Shanghai Medical College, Fudan University, Shanghai, China.

^3.^ Department of Hepatobiliary Surgery, The First Affiliated Hospital of Wannan Medical College, Wuhu, China.

^4.^ Fudan University-the People's Hospital of Rugao Joint Research Institute of Longevity and Ageing, Jiangsu, China.

^5^. Tongji University Cancer Center, Shanghai Tenth People’s Hospital, School of Medicine, Tongji University, Shanghai, China.

**Supplementary materials and methods**

*Cell lines and reagents*

All cell lines were obtained from Cell Bank of Type Culture Collection of Chinese Academy of Sciences (Shanghai, China), and cultured in Dulbecco’s Minimum Essential Medium (DMEM) supplemented with 10% fetal bovine serum (St. Louis, MO, USA) at 37 °C in a humidified atmosphere containing 5% CO2.

*Western blotting*

HCC tissues or cell lines were homogenized in sodium dodecyl sulfate (SDS) sample buffer (10% Glycerol, 2% SDS, 0.01% bromophenol blue, 1.25% 2-beta-mercaptoethanol, 25 mM Tris–HCl, pH 6.8) with ULTRA-TURRAX (IKA, Germany) at 4 °C. Protein concentration was determined with the Quick Start™ Bradford protein assay kit (Bio-Rad, USA). 10μg of total protein extracts was loaded on 10% SDS-PAGE, and transferred to 0.45 μm Polyvinylidene Fluoride (PVDF) membranes (Millipore, USA) using an electro-blotting apparatus (Bio-Rad, USA). Membranes were incubated with antibodies at 4°C overnight before further incubation with Horseradish Peroxidase (HRP)-conjugated secondary antibodies (Proteintech, China). Signal detection used the Immobilon™ Western Chemiluminescence HRP substrate kit (Millipore, USA). Images were obtained with the ImageQuant™ LAS-4000 (Amersham Biosciences, GE, USA), and quantified using the ImageQuant™ TL software (version 7.0, Amersham Biosciences, GE, USA). The diluted primary antibodies were as follows: B4GALNT1 (Proteintech, #13396-1-AP), ERK1/2 (CST #9102), pERK1/2 (CST #4370), JNK (CST #9252), phospho-c-Jun N-terminal kinase (pJNK) (CST #4668), p38 (CST #9212), phospho-p38 (pp38) (CST #4191).

*Co-IP*

Total protein was extracted from the cells using immunoprecipitation lysis buffer (20 mmol/L Tris-HCl, pH 7.6; 150 mmol/L NaCl; 1 mmol/L ethylene diamine tetraacetic acid (EDTA); 0.5% NP-40; 10% glycerol; 1 mmol/L phenylmethanesulfonyl fluoride). The extracted protein was pretreated with protein A/G plus-agarose beads (Millipore) overnight at 4°C. Subsequently, these mixtures were incubated with HES4 (abcam, ab272675) or MAFG (abcam, ab154318) antibody at 4°C for 24 h, respectively. Afterwards, the collected beads were washed three times using washing buffer (50 mmol/L Tris-HCl, pH 7.6; 300 mmol/L NaCl; 1 mmol/L EDTA; 0.5% NP-40; 10% glycerol), and the post-elution beads were boiled in the loading buffer, resolved on SDS-PAGE. Immunoblotting was then performed.

*Enzyme-linked immunosorbent assay*

The culture media was concentrated using Amicon Ultra-15 units (Millipore, Merk, USA), and the concentrating rate (concentrated volume/input volume) was recorded for final calculation. To measure the levels of MIF and SPP1 in the concentrated media, enzyme-linked immunosorbent assay (ELISA) was performed using Human MIF Quantikine ELISA Kit and Human Osteopontin (SPP1) Quantikine ELISA Kit (R&D SYSTEMS, USA). All operations were performed according to the instructions provided by the manufacturer. Secreting levels of MIF and SPP1 in the original culture media were calculated and normalized to pg/ml.

*qPCR*

Total RNA of samples was purified using TRIzol (Invitrogen, Carbad, CA, USA), and then transcribed to cDNA using PrimeScript RT reagent Kit (Takara, Tokyo, Japan). Real-time PCR was performed with the cDNA production using SYBR Premix Ex Taq (Takara, Tokyo, Japan) on ABI StepOne Plus (Applied Biosystems, USA). Glyceraldehyde-3-phosphate dehydrogenase (GAPDH) was used as an internal control. All the primers used were listed below:

B4GALNT1 forward, 5’-CAGAAACAAGTCCGAGCTATTGA-3’;

B4GALNT1 reversed, 5’-GAGGGGCTGAACTTCCACAC-3’;

GAPDH forward, 5’-GAGTCAACGGATTTGGTCGT-3’;

GAPDH reversed, 5’-TTGATTTTGGAGGGATCTCG-3’;

MIF forward, 5’ CAGCCCGGACAGGGTCTAC-3’;

MIF reversed, 5’-TCTTAGGCGAAGGTGGAGTTG-3’;

SPP1 forward, 5’ GAAGTTTCGCAGACCTGACAT-3’;

SPP1 reversed, 5’- GTATGCACCATTCAACTCCTCG-3’.

*Small interfering RNA (siRNA)*

Synthesized siRNA oligonucleotides were purchased from Biotend (China). After dissolution and resuspension under RNase-free condition, the sense and antisense RNA oligonucleotides was mixed and annealed for 1 min at 90 °C, followed by incubation for 1 hour at 37 °C. Then, double stranded siRNA was transfected in PLC/PRF/5 cells, and these cells were harvested 72 hours after transfection for further qPCR and WB analysis. All the siRNAs used were listed below:

siRNA1-B4GANT1, guide sequence: AAAAAAUGCAGAAUCAUGCCC; passenger sequence: GCAUGAUUCUGCAUUUUUUCC;

siRNA2-B4GANT1, guide sequence: AAACAUAACUCCUAGUAUGCU; passenger sequence: CAUACUAGGAGUUAUGUUUGC;

siRNA2-control, guide sequence: AGUUCAACGACCAGUAGUCdTdT; passenger sequence: GACUACUGGUCGUUGAdTdT.

*IHC*

IHC analysis was applied on tissue microarray using Dako REAL EnVision Detection System (Dako, Denmark) according to the manufacture instruction. Antibodies against B4GALNT1 and CD163 were used to quantify the relative levels of B4GALNT1 in tumor cells and the number of CD163+ macrophages. Hematoxylin was used for counterstaining. Immunohistochemical scoring was determined as previous described [1]. The staining intensity was scored as 0 for negative; 1 for weak; 2 for moderate weak; 3 for moderate strong and 4 for strong. The score for the stained area was set as 0 for 0%–33%; 1, 33%–66%; and 2, 66%–100%. The final staining score was obtained by multiplying staining intensity score with staining area score, and the results are a series number ranging from 0 to 8.

*Luciferase assays*

Luciferase assays were performed in HEK293T cells with the responsive reporter MIF-Luc or SPP1-Luc. MIF-Luc or SPP1-Luc reporters (50 ng) were transfected together with Renilla (100 ng) to normalize for transfection efﬁciency with Dual-Luciferase Reporter Assay System (Promega, USA) in 24-well tissue culture plates. HES4 plasmids were co-transfected with 50, 100, and 200 ng for dose, respectively. DNA content in all samples was kept uniform by adding vector plasmid up to 350 ng per well. 72 hours after transfection, cells were lysed and luciferase reporter assay was performed using the dual luciferase reporter assay kit from Promega as described. Each sample was transfected in triplicate and each experiment was repeated at least three times independently.

*Transwell assay*

The recruitments on macrophages and T cells were assessed using 8-μm transwell chamber (Milliporem, USA) in a 12-well plate. The bottom of the transwell chamber was coated with BD Matrigel Basement Membrane Matrix (BD Biosciences, USA). Macrophages or T cells were added into the upper chamber containing basic culture medium without serum, and the lower chamber was filled with the serum-free culture medium of tumor cells, or with tumor cells in serum-free culture medium. The infiltration was determined 48 h later. For macrophages, cells on the upper side of the chamber were removed from the surface of the membrane by scrubbing, and cells on the lower surface of the membrane were fixed with 4% paraformaldehyde and stained with 0.1% crystal violet. The number of infiltrating cells was counted in five randomly selected microscopic fields of each filter. For T cells, the infiltrating rates were calculated by dividing the total cell number by the infiltrated numbers in lower chamber.

*FACS*

Single-cell suspensions from xenograft tumors were derived through mechanical separation and passage through a 70 μm filter screen. Tumor suspensions were stained with PerCP anti-mouse CD45, PE anti-mouse CD4 and APC anti-mouse CD8a, or PE anti-mouse CD11b and APC anti-mouse CD168 in the dark for 30 min at 4 °C. All samples were acquired on BD FACSVerse (BD Biosciences) and were analyzed using FlowJo (FlowJo 7.6).

*Single-cell RNA analysis*

Single-cell sequencing data of HCC tumor tissues were analyzed by applying "Seurat" package (4.0.0). Briefly, data of GSE125449 and GSE149614 were loaded as Seurat objects. After normalization, 2000 highly variable genes were used to scaling. Then, all cells were clustered using the "KNN" method at a resolution of 0.5, and the final sub-clustering patterns were adjusted according to previous reports [2, 3]. In addition, malignant cells were picked out using copy number karyotyping methods with the “copycat” package (1.1.0). The intercellular communication networks were determined with the “CellChat” package (1.1.3). And the regulatory activities of TFs were investigated using the “pySCENIC” package (0.11.2) [4, 5]. In pySCENIC analysis, target genes with a low positive correlation (*p* < 0.03) in each TF module were removed finally, and the activity of each transcriptional regulon was computed as the AUC scores and regulon specificity scores (RSS).

*Differential Analysis*

The differential profiles of B4GALNT1-associated genes and pathways were analyzed using the “limma” package (3.20.9) between B4GALNT1-positive and B4GALNT1-negative patients or tumor cells. Adjusted *p* < 0.05 were employed as standard screening criteria.

*GSEA and GSVA*

GSEA and GSVA were used to evaluate gene-related functions. The GO C5 gene set from the Molecular Signature Database (MSigDB) was downloaded and used for the annotation. The “clusterProfiler” package (4.4.4) was used for GSEA analysis and the “GSVA” package (1.44.2) was used for GSVA analysis. Adjusted *p*-value was used to determine whether there were significant differences.

*Immune Cell Infiltration*

To uncover the correlations between B4GALNT1 expression and tumor-infiltrating immune profiles, eight tools including XCELL R version, XCELL online version [6], TIMER [7], QUANTISEQ [8], MCPcounter [9], EPIC [10], CIBERSORT [11] and CIBERSORTx [12] were utilized to evaluate the immune infiltrating situation, and the correlations were analyzed with the methods of Spearman.

**References**

1. Song SS, Peng PK, Tang ZQ, Zhao JJ, Wu WC, Li HJ, et al. Decreased expression of STING predicts poor prognosis in patients with gastric cancer. Sci Rep-Uk. 2017;7. http://doi.org/10.1038/srep39858.

2. Lu YM, Yang AQ, Quan C, Pan YW, Zhang HY, Li YF, et al. A single-cell atlas of the multicellular ecosystem of primary and metastatic hepatocellular carcinoma. Nat Commun. 2022;13(1). http://doi.org/10.1038/s41467-022-32283-3.

3. Ma LC, Hernandez MO, Zhao YM, Mehta M, Tran B, Kelly M, et al. Tumor Cell Biodiversity Drives Microenvironmental Reprogramming in Liver Cancer. Cancer Cell. 2019;36(4):418-+. <http://doi.org/10.1016/j.ccell.2019.08.007>.

4. van de Sande B, Flerin C, Davie K, De Waegeneer M, Hulselmans G, Aibar S, et al. A scalable SCENIC workflow for single-cell gene regulatory network analysis. Nat Protoc. 2020;15(7):2247-76. <http://doi.org/10.1038/s41596-020-0336-2>.

5. Ferlay J, Shin HR, Bray F, Forman D, Mathers C, Parkin DM. Estimates of worldwide burden of cancer in 2008: GLOBOCAN 2008. Int J Cancer. 2010;127(12):2893-917. <http://doi.org/10.1002/ijc.25516>.

6. Aran D, Hu ZC, Butte AJ. xCell: digitally portraying the tissue cellular heterogeneity landscape. Genome Biol. 2017;18. http://doi.org/10.1186/s13059-017-1349-1.

7. Li B, Severson E, Pignon JC, Zhao HQ, Li TW, Novak J, et al. Comprehensive analyses of tumor immunity: implications for cancer immunotherapy. Genome Biol. 2016;17. http://doi.org/10.1186/s13059-016-1028-7.

8. Finotello F, Mayer C, Plattner C, Laschober G, Rieder D, Hackl H, et al. Molecular and pharmacological modulators of the tumor immune contexture revealed by deconvolution of RNA-seq data (vol 11, 34, 2019). Genome Med. 2019;11. http://doi.org/10.1186/s13073-019-0655-5.

9. Becht E, Giraldo NA, Lacroix L, Buttard B, Elarouci N, Petitprez F, et al. Estimating the population abundance of tissue-infiltrating immune and stromal cell populations using gene expression (vol 17, 218, 2016). Genome Biol. 2016;17. http://doi.org/10.1186/s13059-016-1113-y.

10. Racle J, de Jonge K, Baumgaertner P, Speiser DE, Gfeller D. Simultaneous enumeration of cancer and immune cell types from bulk tumor gene expression data. Elife. 2017;6. http://doi.org/10.7554/eLife.26476.

11. Newman AM, Liu CL, Green MR, Gentles AJ, Feng WG, Xu Y, et al. Robust enumeration of cell subsets from tissue expression profiles. Nat Methods. 2015;12(5):453-. <http://doi.org/10.1038/Nmeth.3337>.

12. Newman AM, Steen CB, Liu CL, Gentles AJ, Chaudhuri AA, Scherer F, et al. Determining cell type abundance and expression from bulk tissues with digital cytometry. Nat Biotechnol. 2019;37(7):773-. <http://doi.org/10.1038/s41587-019-0114-2>.

**Supplementary figures**


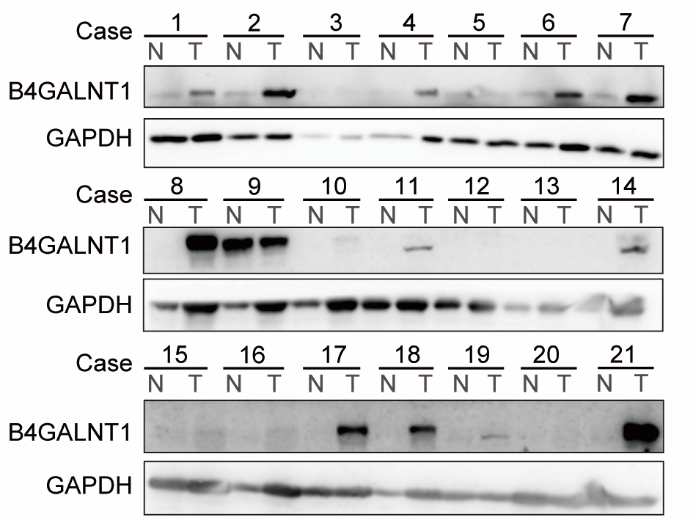


**Figure S1.** Western blot analysis was applied to detect relative protein expression level of B4GALNT1 in 21 pairs of HCC tissues. GAPDH was used as control. T, HCC tumor tissue; N, adjacent non-tumor tissue.


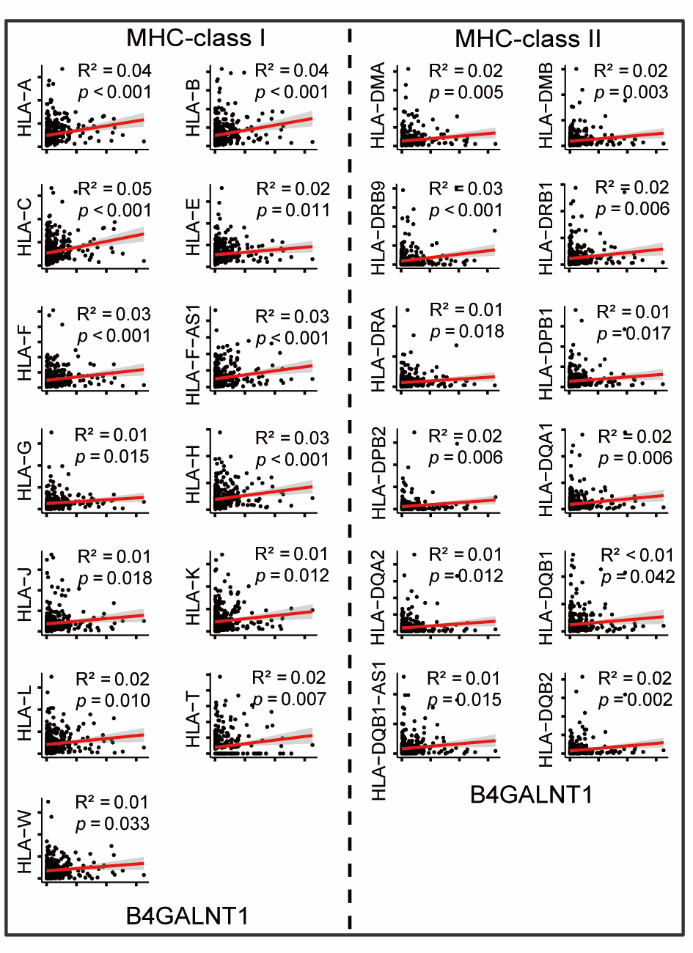


**Figure S2.** Expressional correlation between B4GALNT1 and the HLA genes including MHC-class I (left) and MHC-class II (right) in TCGA-LIHC.


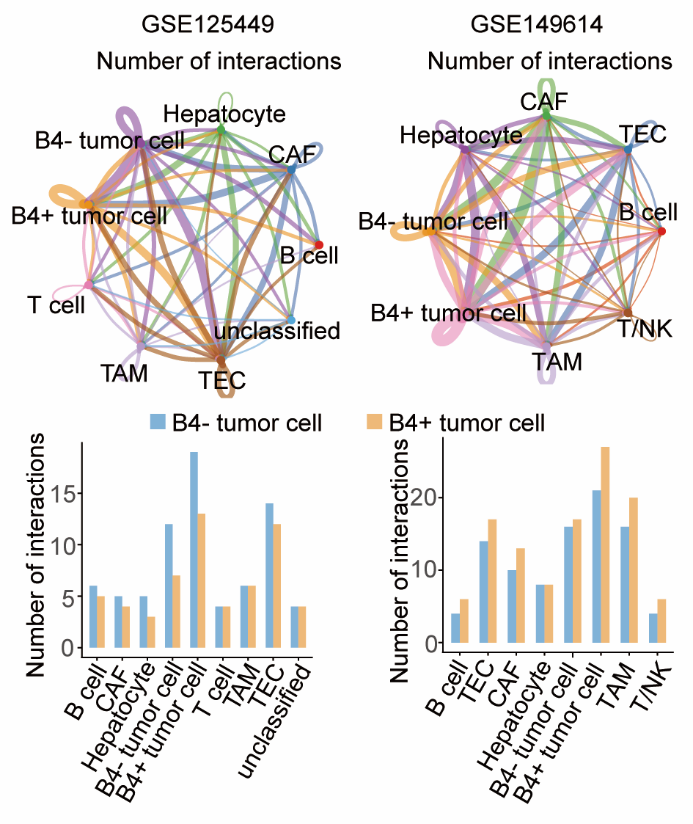


**Figure S3.** CellChat analysis for the cross-talk between different cell types. The cross-talk counts between B4GALNT1-positive malignant cells, B4GALNT1-negative malignant cells, TAMs, T-cells and other cell types were determined in GSE125449 (Up left) and GSE149614 (Up right). And the CellChat differences between B4GALNT1-positive and B4GALNT1-negative malignant cells were also analyzed (down).


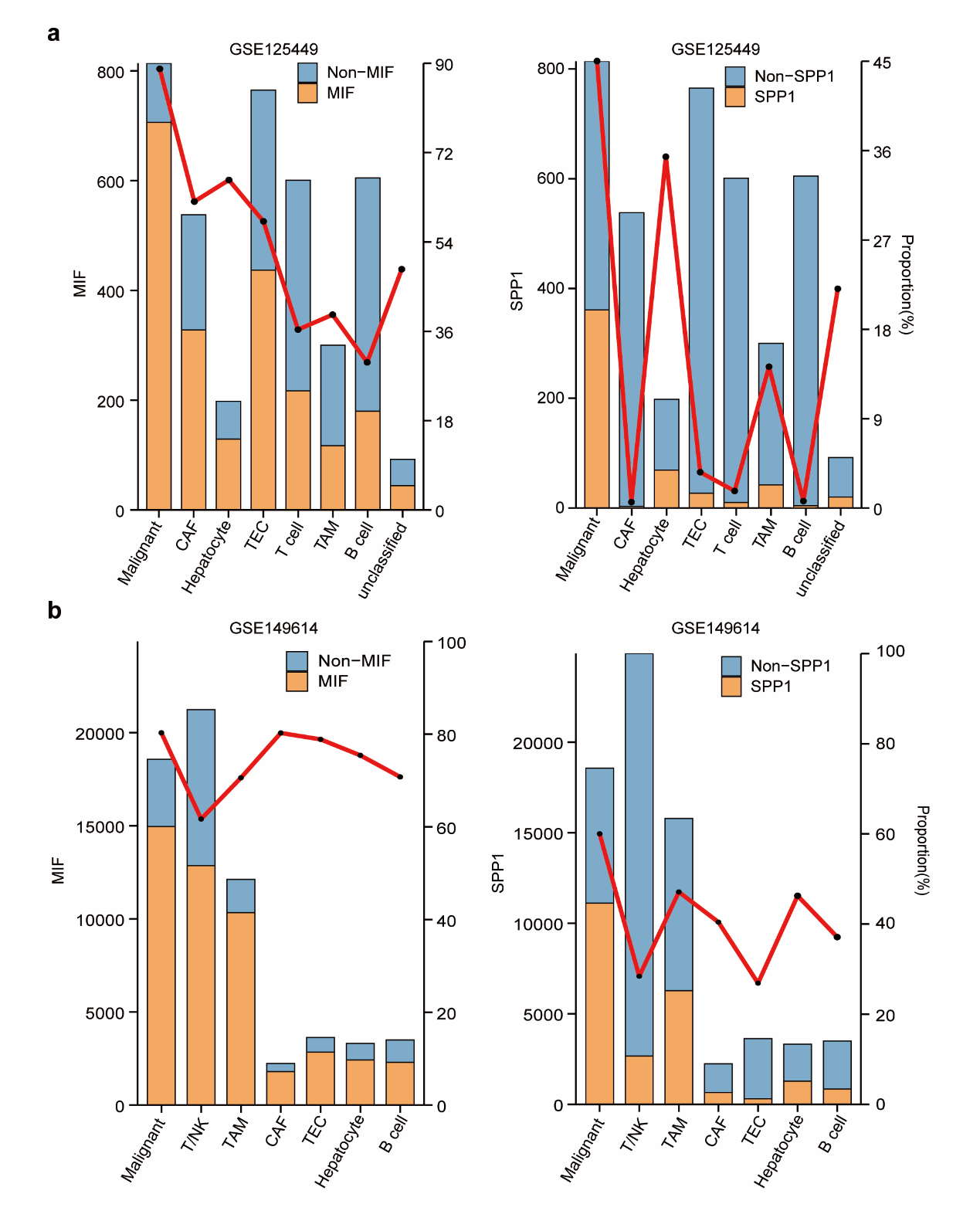


**Figure S4.** (a-b) Numbers (bar plot, left y-axis) and proportions (line chart, right y-axis) of different cell types in tumor tissues from GSE125449 (a) and GSE149614 (b) of MIF (left) and SPP1 (right) positive cells in these clusters. Orange, B4GALNT1-positive (B4+) tumor cell; Blue, B4GALNT1-negative (B4-) tumor cell.


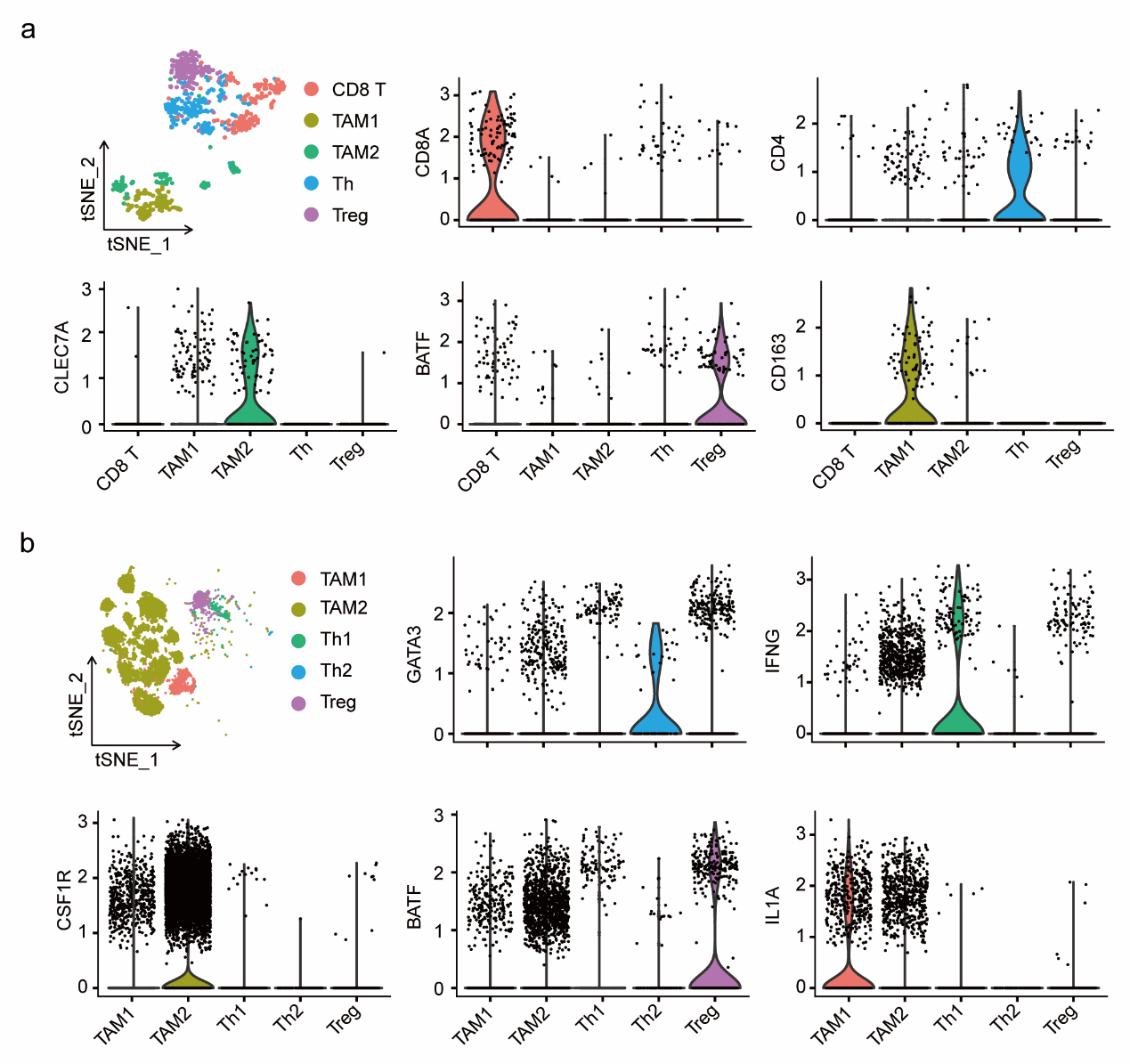


**Figure S5.** (a-b) TSNE (up left) dimensionality reduction was performed to visualize the 5 clusters of GSE125449 (a) and GSE149614 (b) datasets. For each cluster, violin plots of genes previously reported to be enriched are plotted alongside the TSNE plot.


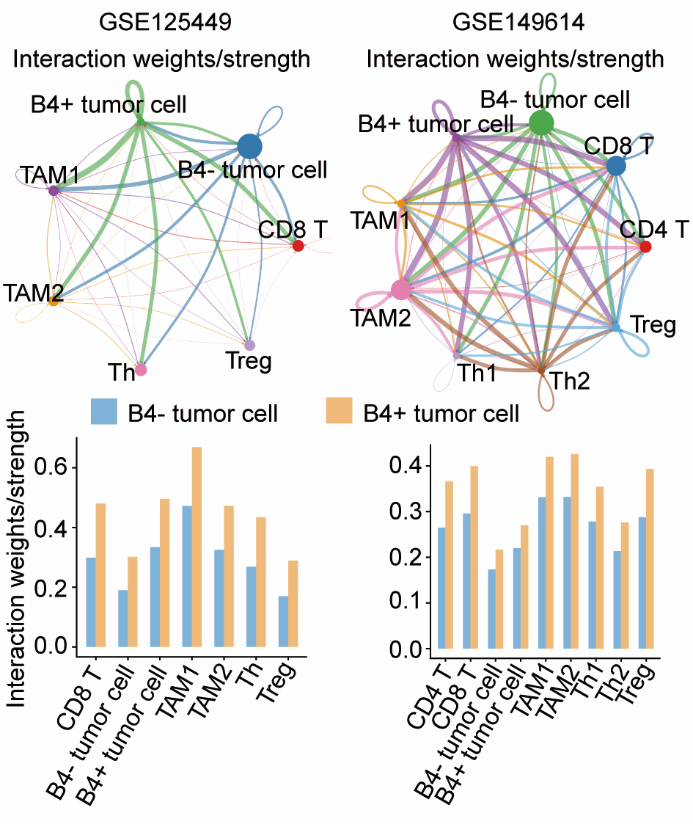


**Figure S6.** CellChat analysis for the cross-talk between different cell types. Cross-talk strengths were determined among B4GALNT1-positive malignant cells, B4GALNT1-negative malignant cells, TAM1, TAM2, Th, Treg and other cell types in GSE125449 (Up left) and GSE149614 (Up right). And the CellChat differences between B4GALNT1-positive and B4GALNT1-negative malignant cells were also analyzed (down).


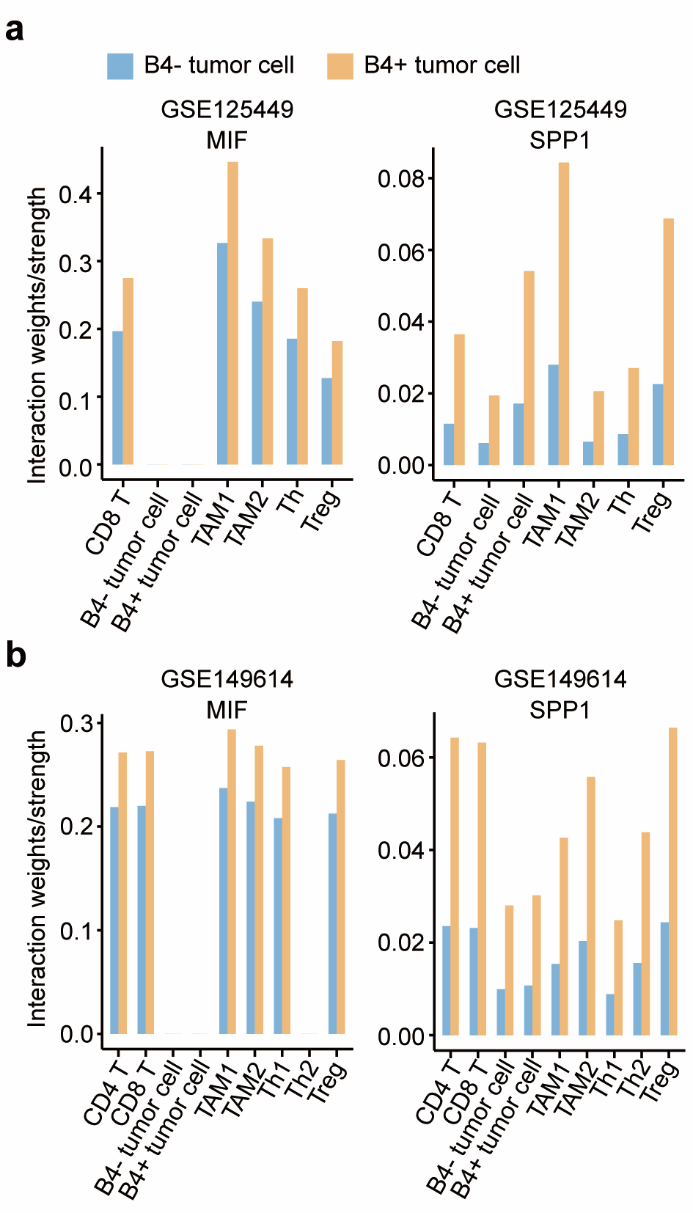


**Figure S7.** (a-b) CellChat differences between B4GALNT1-positive and B4GALNT1-negative malignant cells of MIF (left) and SPP1 (right) pathways were analyzed in GSE125449 (a) and GSE149614 (b).


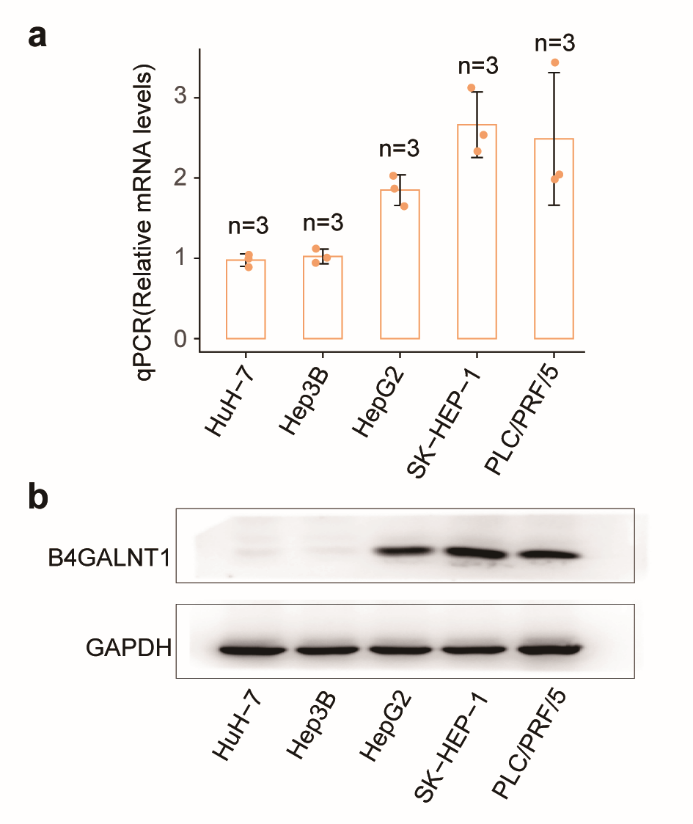


**Figure S8.** (a-b) B4GALNT1 in Huh7, Hep3B, HepG2, SK-HepG2 and PLC/PRF/5 cells determined by qPCR (a) and WB analysis (b).


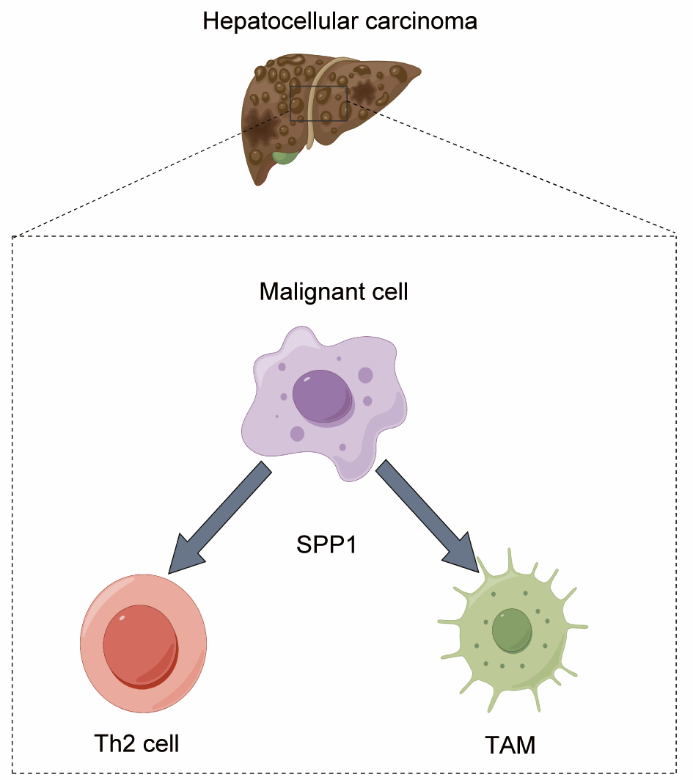


**Figure S9.** The schematic diagram for the immune microenvironment affected by malignant cells (By Figdraw).


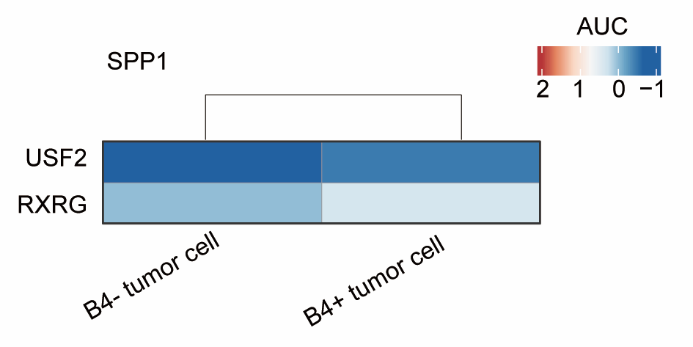


**Figure S10.** Activation differences of the SPP1-associted TFs according to the RcisTarget reference between B4GALNT1-positive and -negative malignant cells.


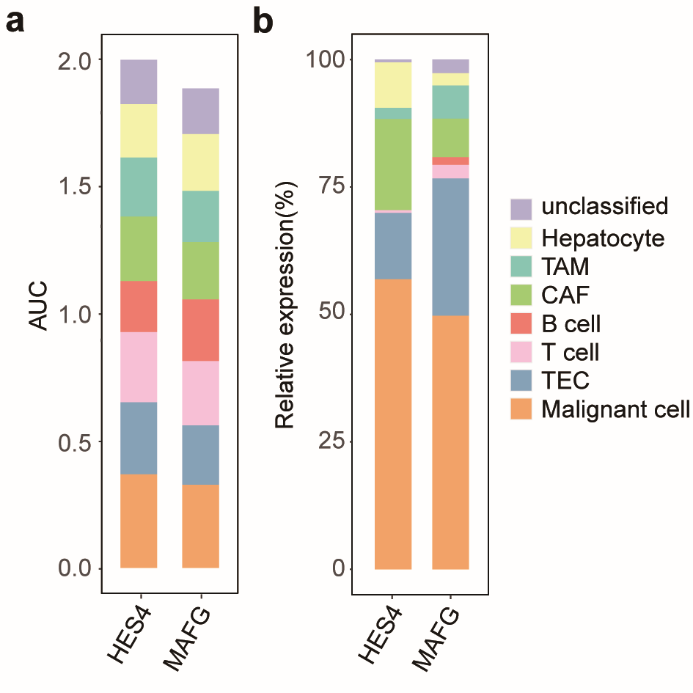


**Figure S11.** (a-b) Activation (a) and relative expression (b) of HES4 and MAFG in different cell types from GSE125449.


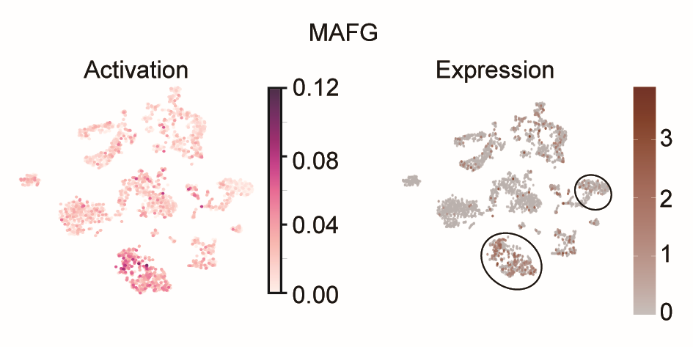


**Figure S12.** Activation (left) and expression (right) distribution of MAFG in different cell types of GSE125449.


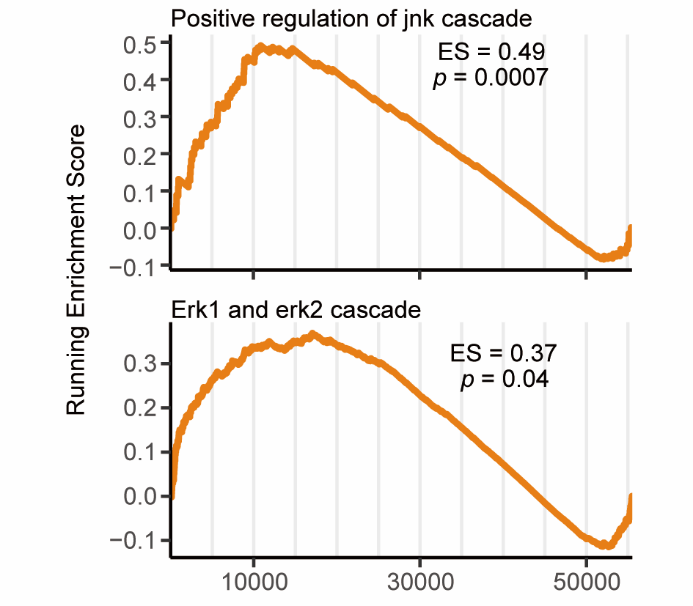


**Figure S13.** Gene set enrichment analysis (GSEA) of B4GALNT1 overexpression correlating with the upregulation of the JNK and ERK signaling pathway.


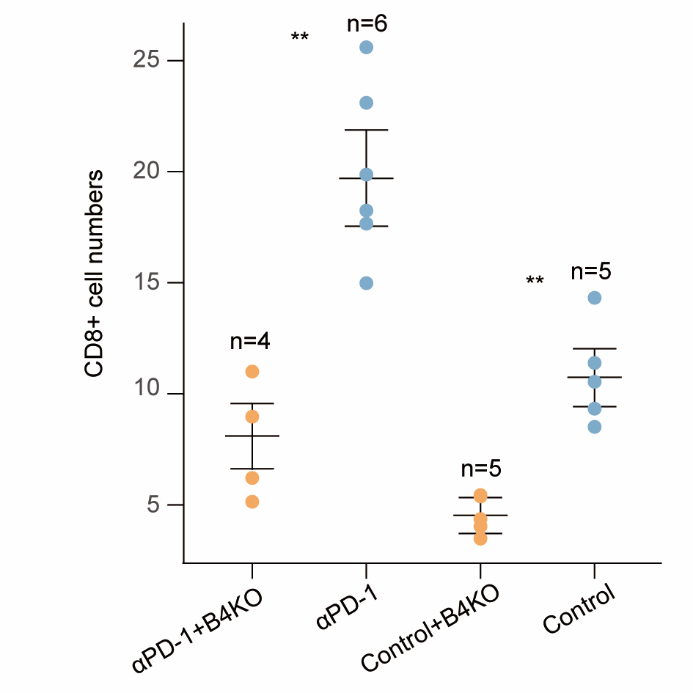


**Figure S14.** Statistical analysis for the numbers of CD8+ T cells in the xenografts under different treatments.


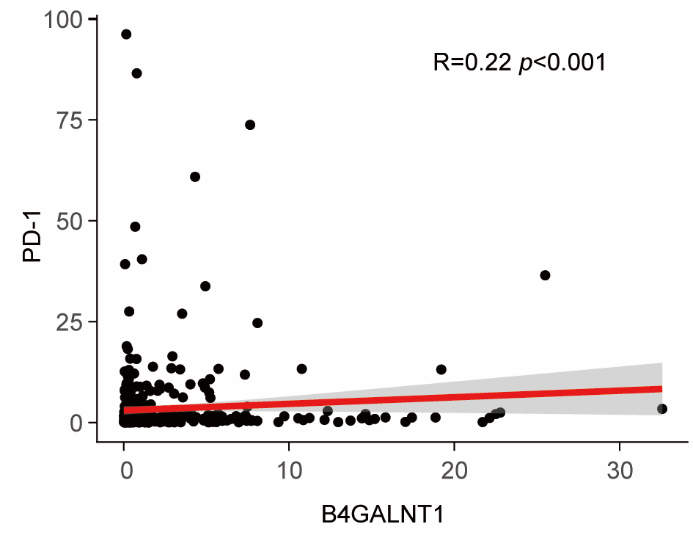


**Figure S15.** Expressional correlation between B4GALNT1 and PDCD1 in TCGA-LIHC.


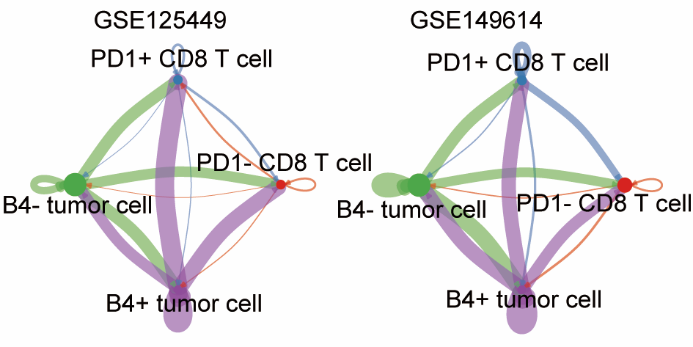


**Figure S16.** CellChat analysis for the cross-talk between different cell types. The cross-talk strengths determined by interaction weight were determined among B4GALNT1-positive malignant cell, B4GALNT1-negative malignant cell, PD1-positive CD8 T cell and PD1-negative CD8 T cell in GSE125449 (left) and GSE149614 (right).

**Full uncropped gels and blots images**

Figure 1c


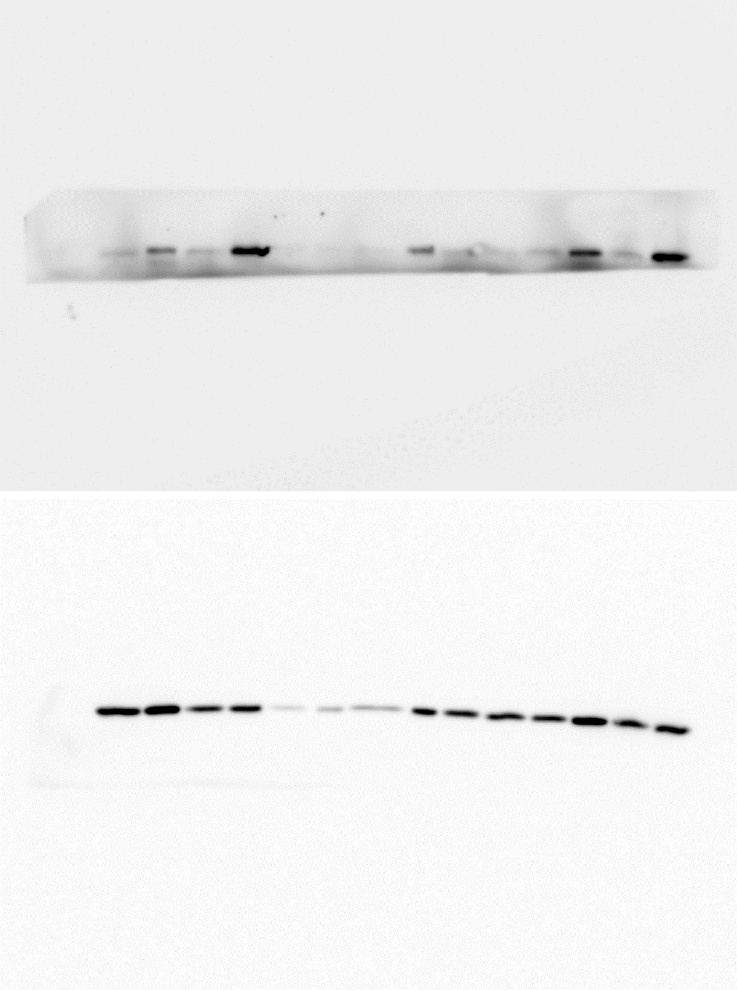

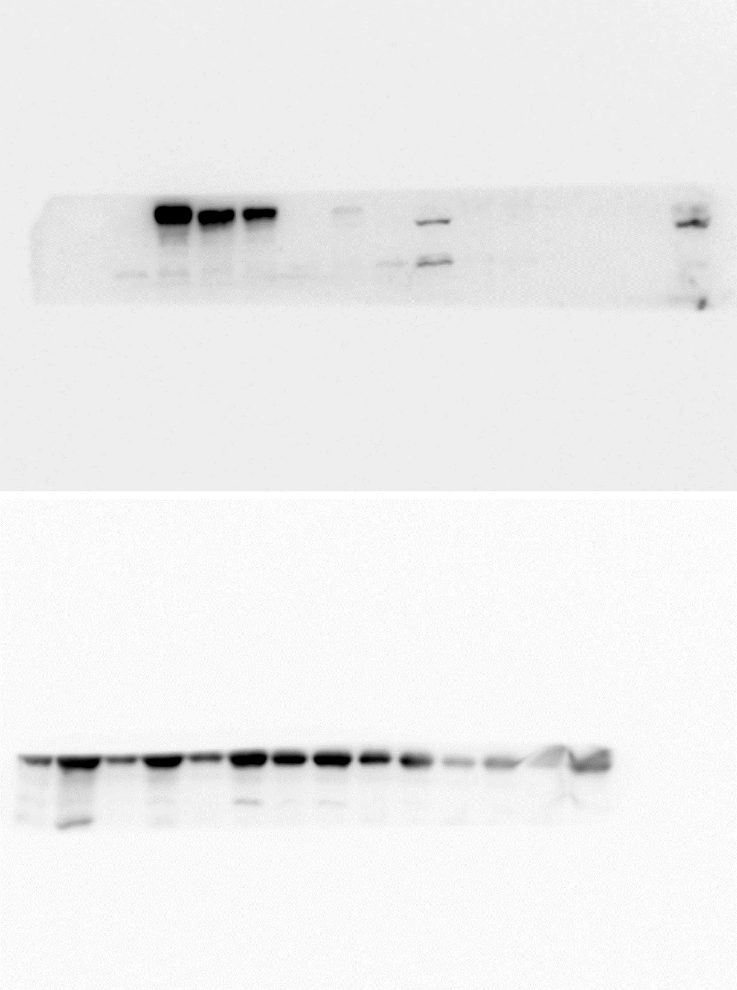

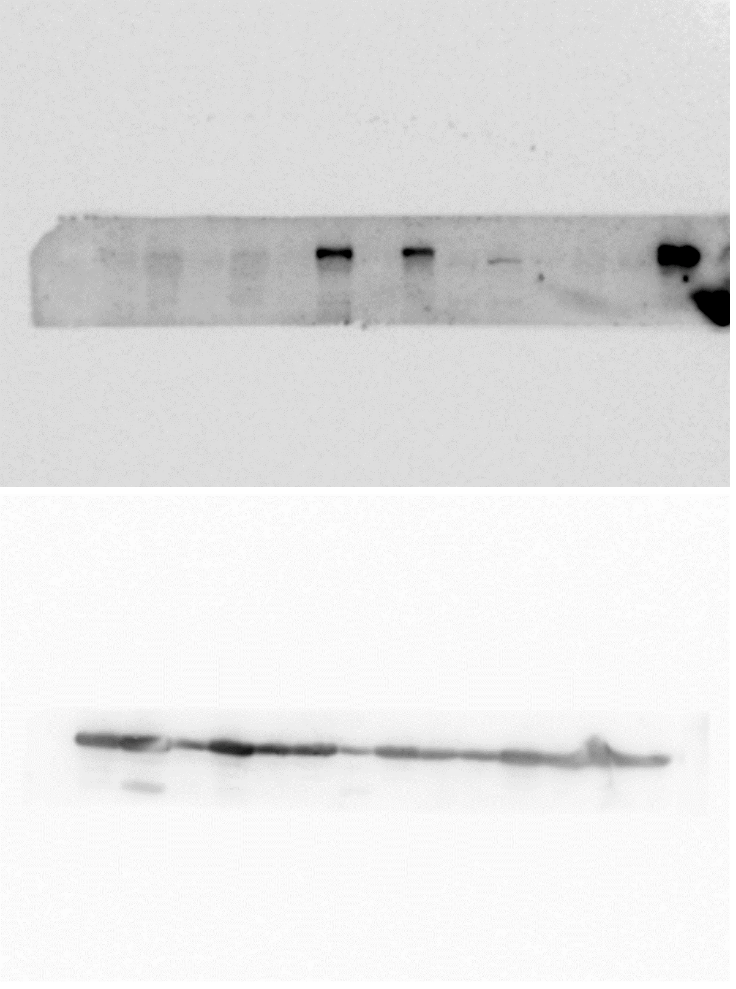


Figure 6i


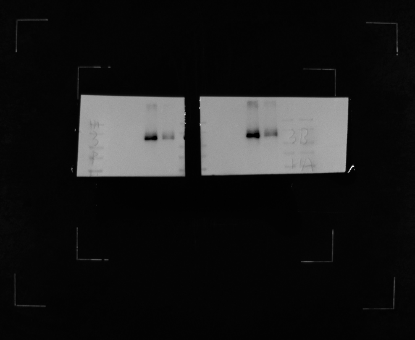

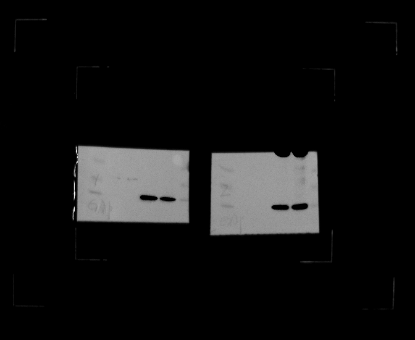


Figure 6l


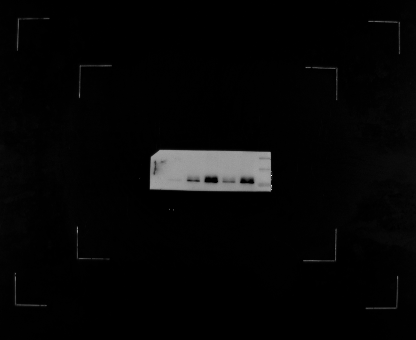

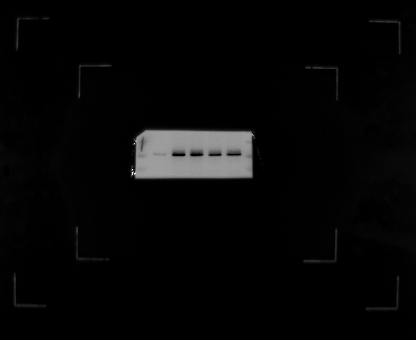


Figure 7i


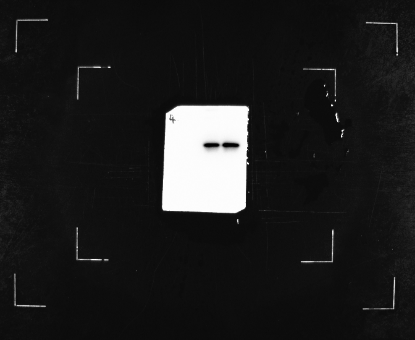

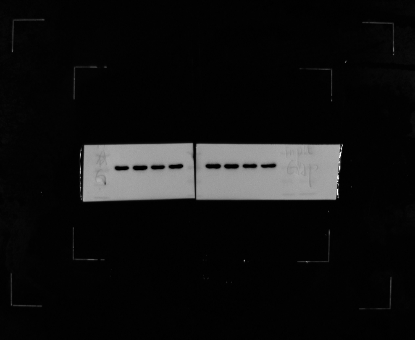


Figure 7j


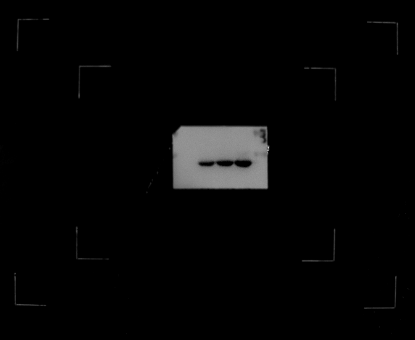

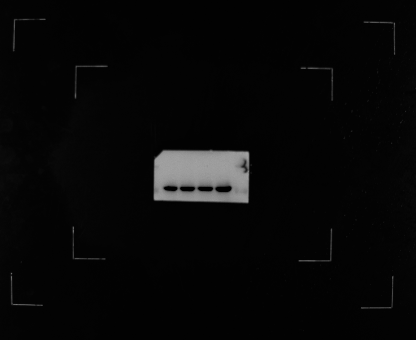


Figure 7k


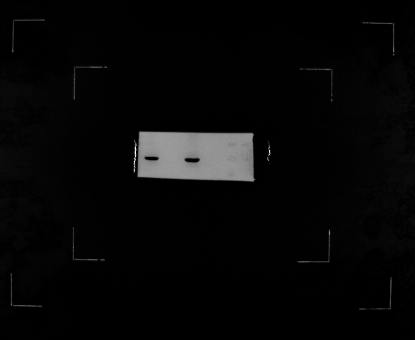

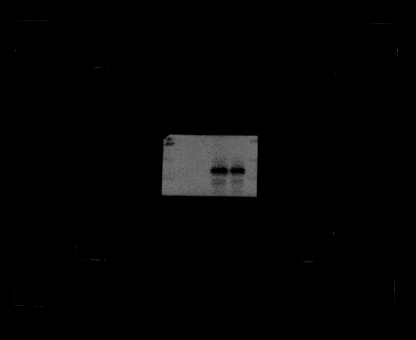

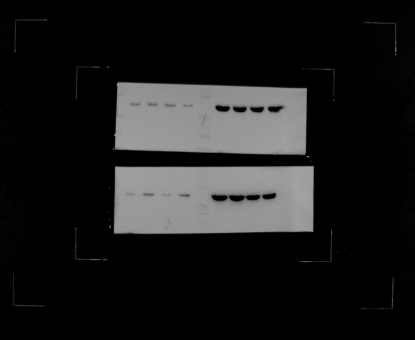


Figure 7l


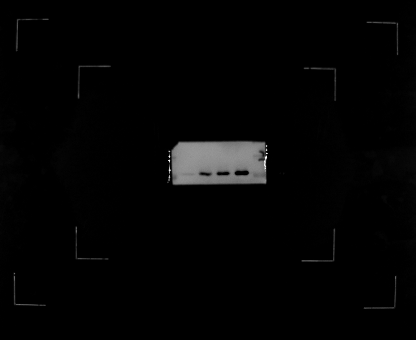


Figure 8a


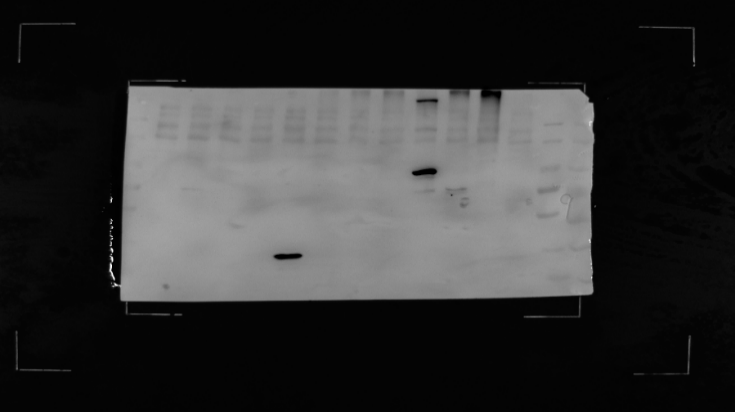

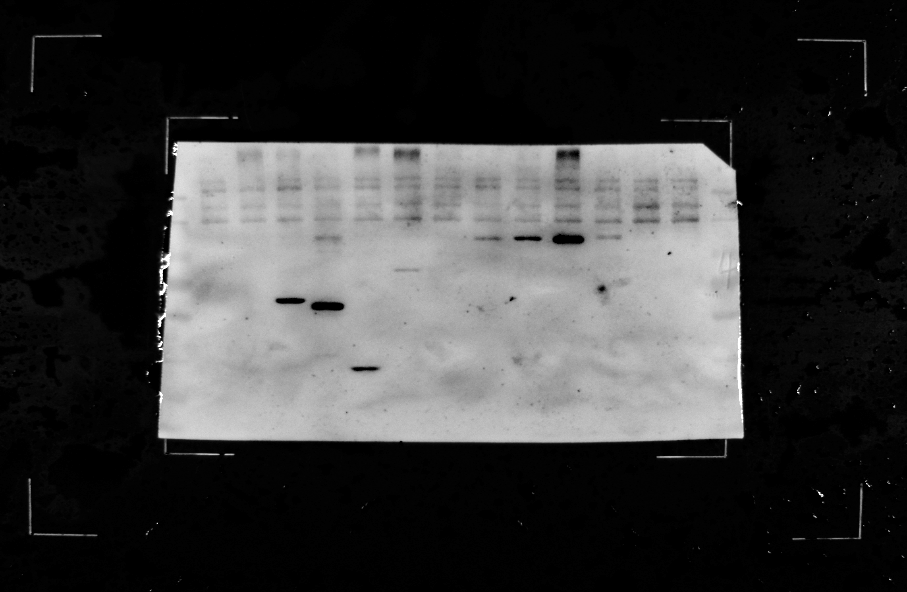

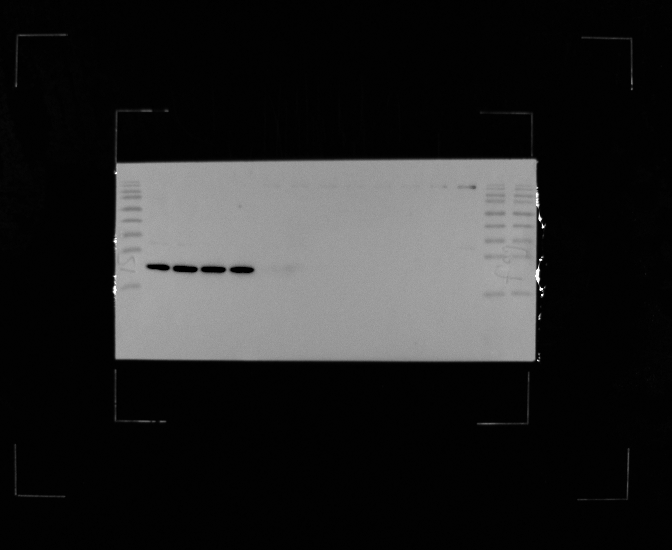

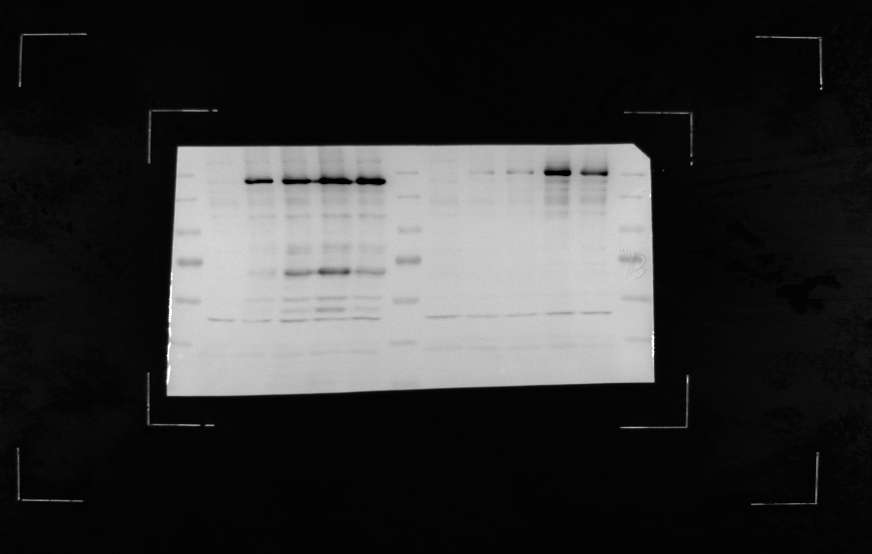


Figure 8b


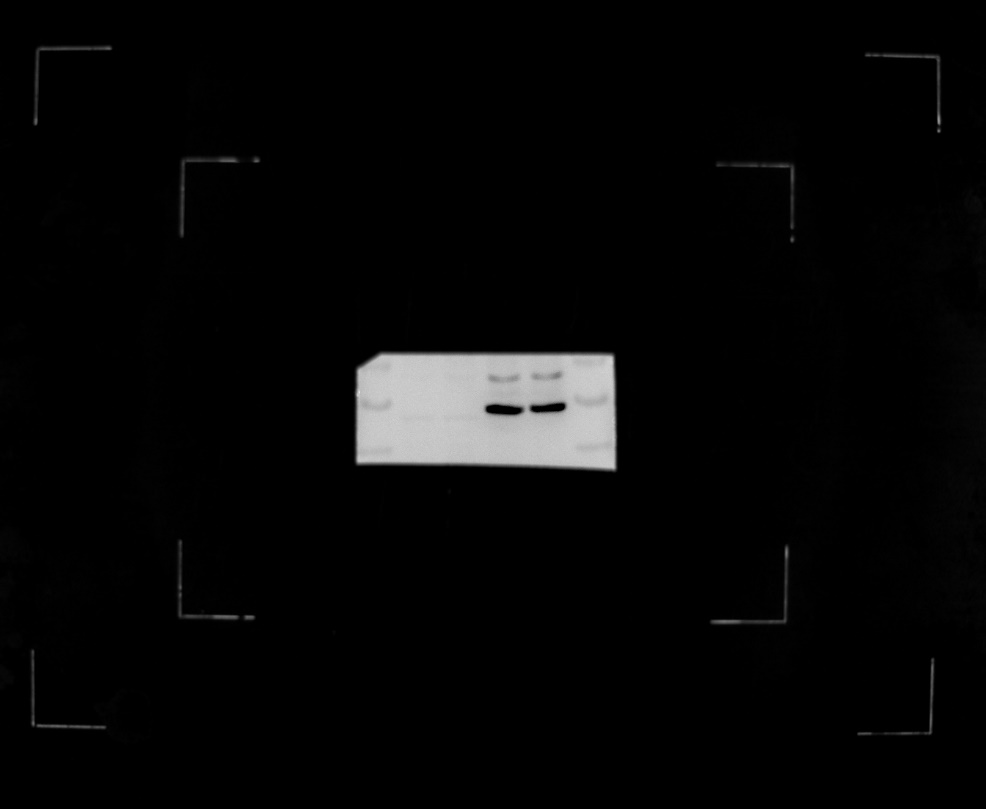

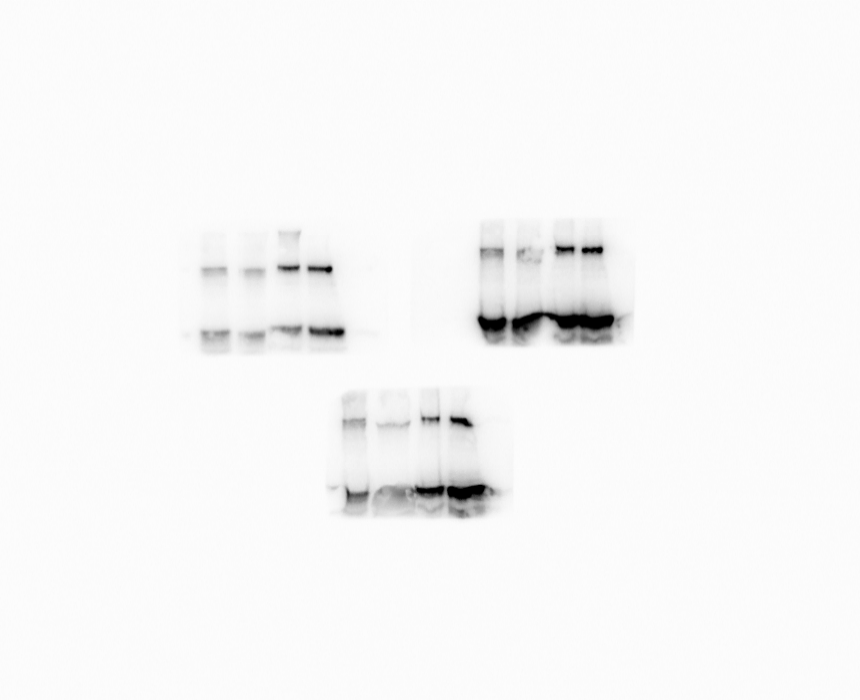

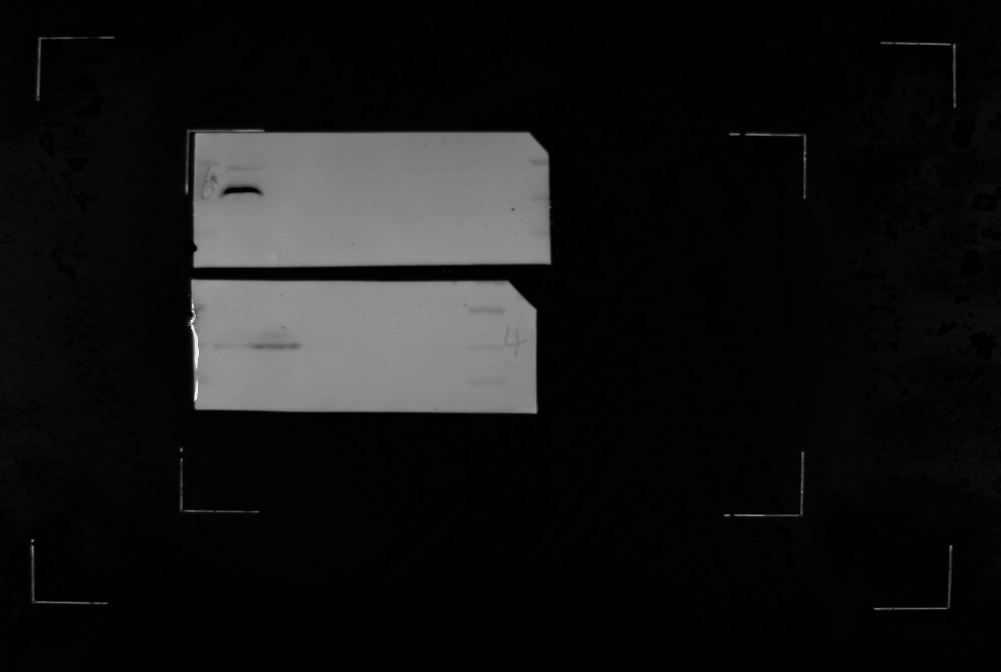

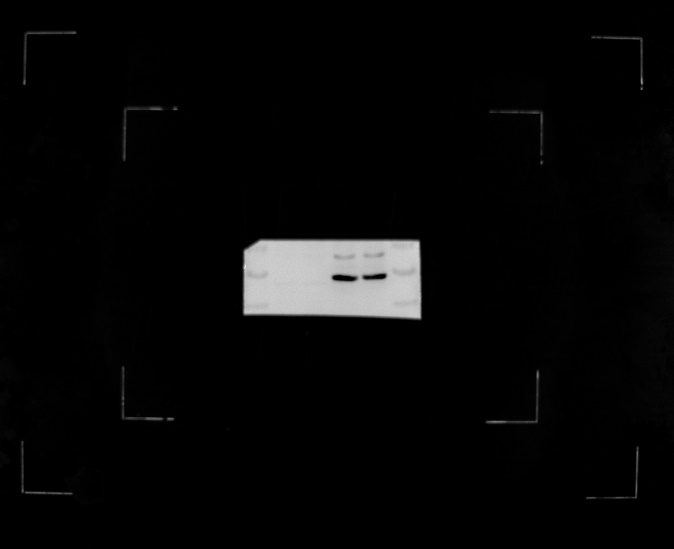

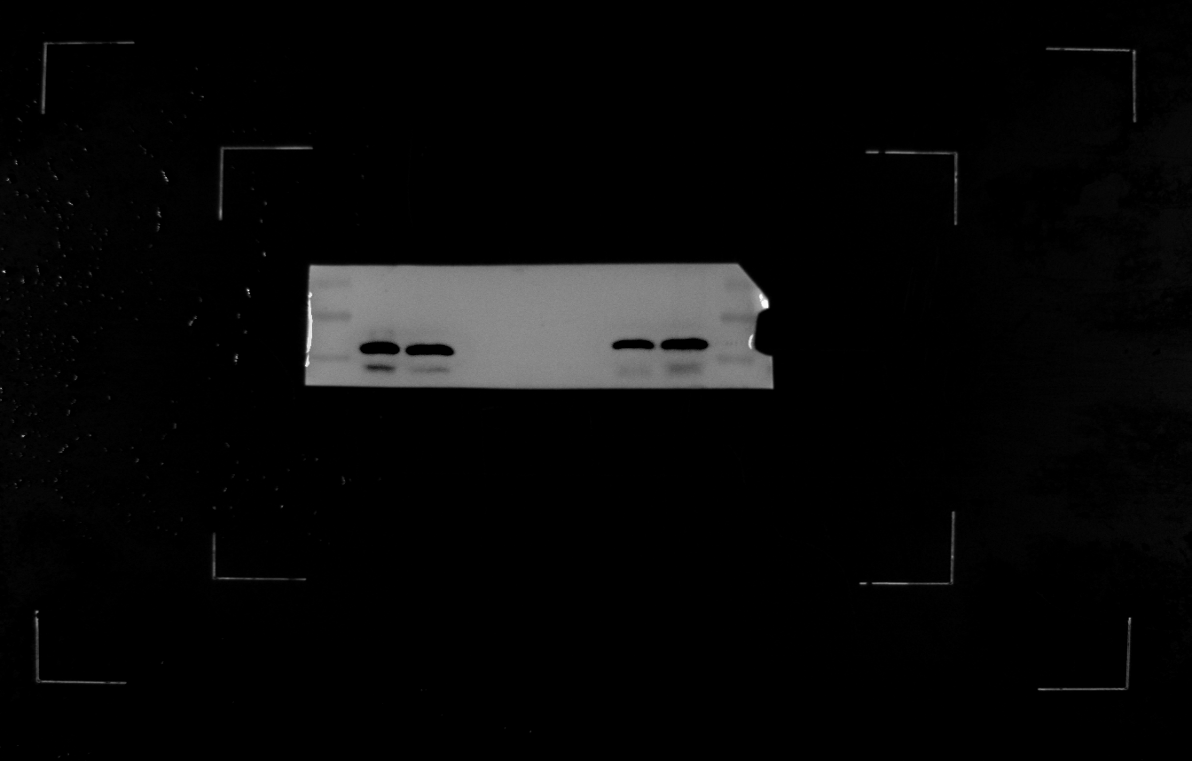


Figure 8c


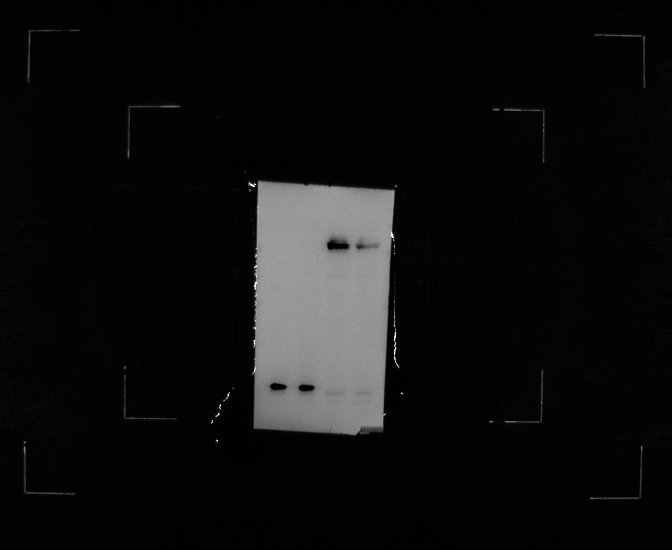

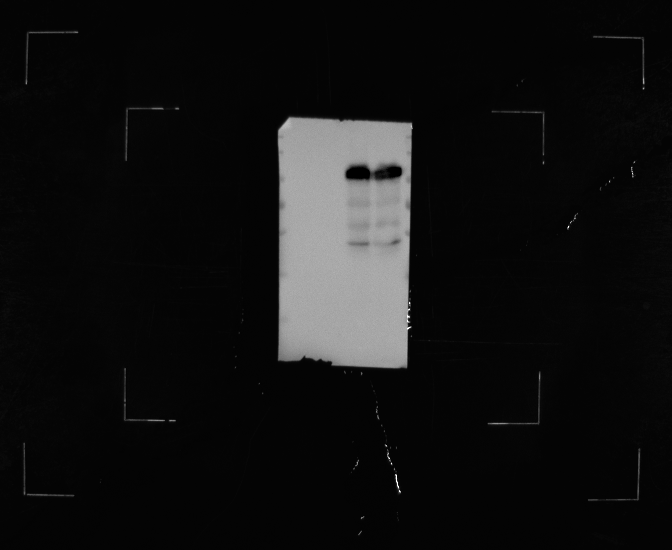

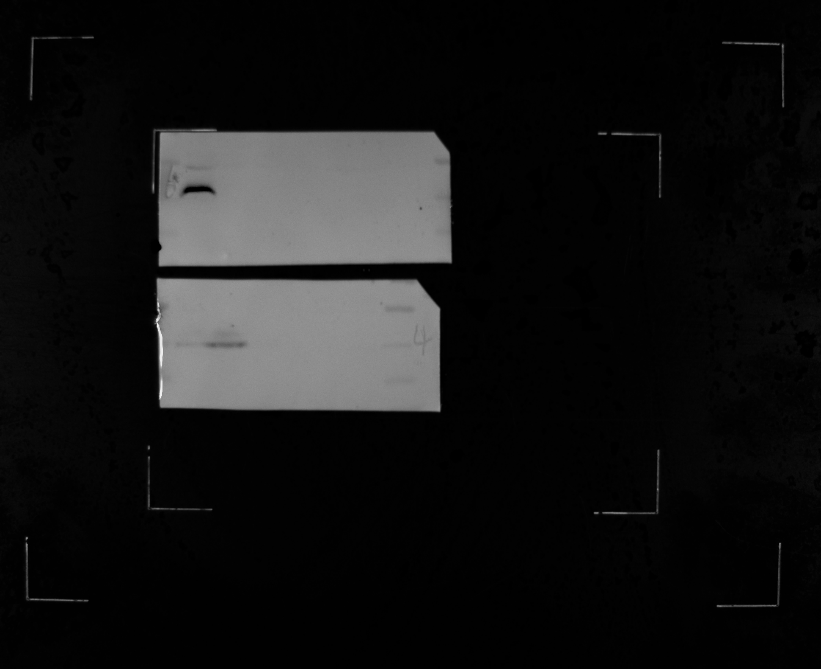

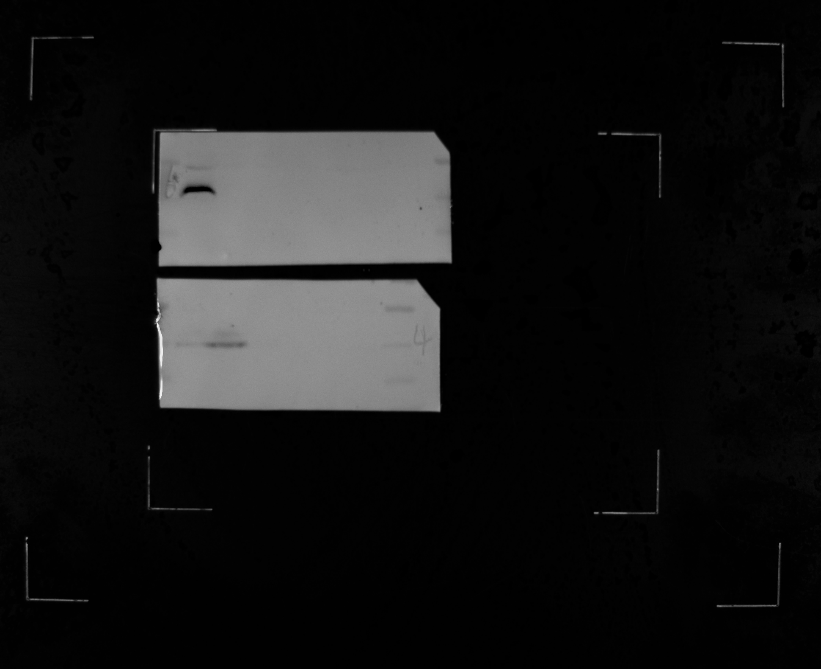

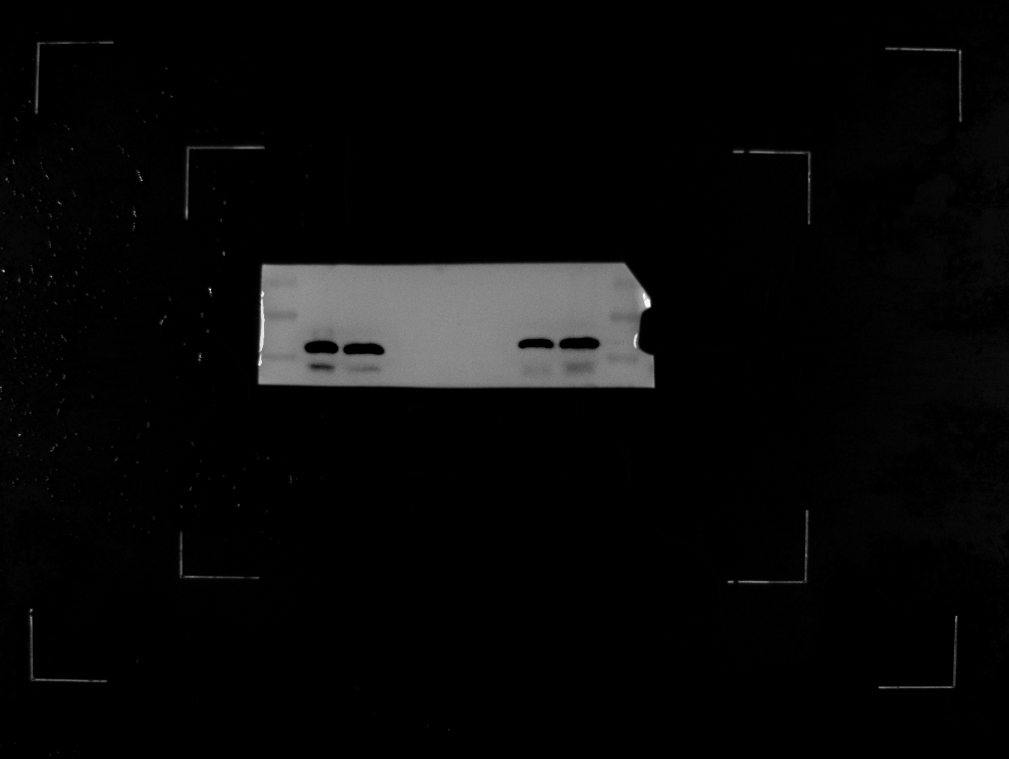


Figure 8g


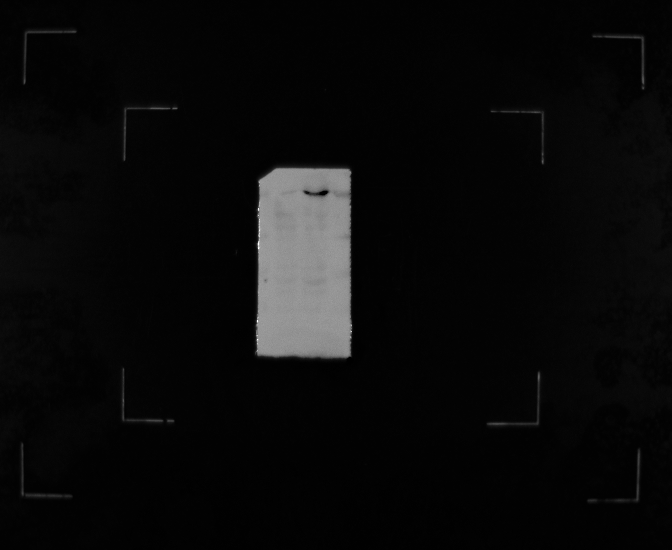

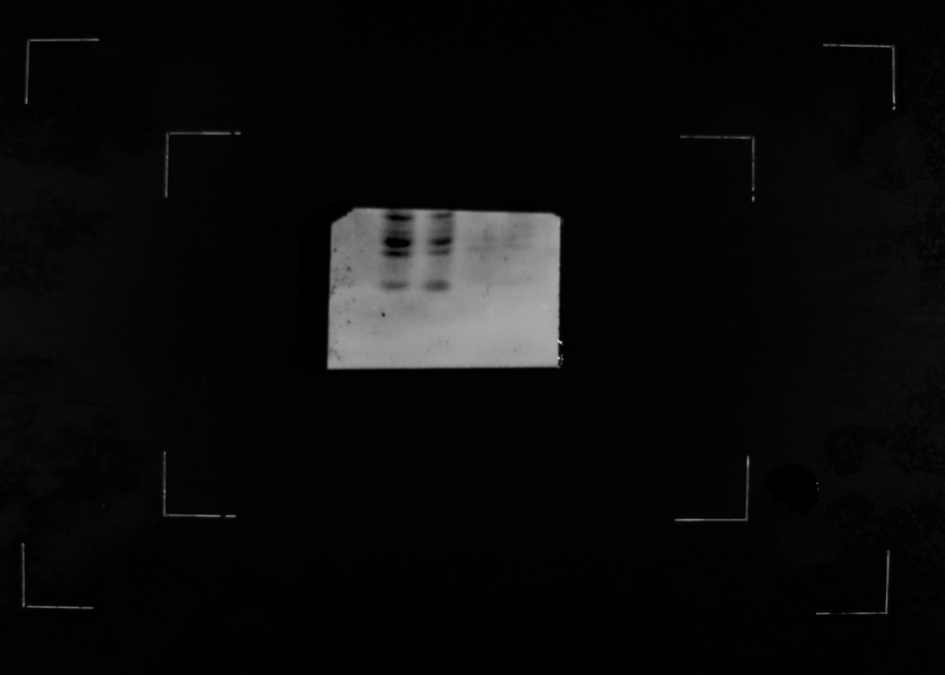

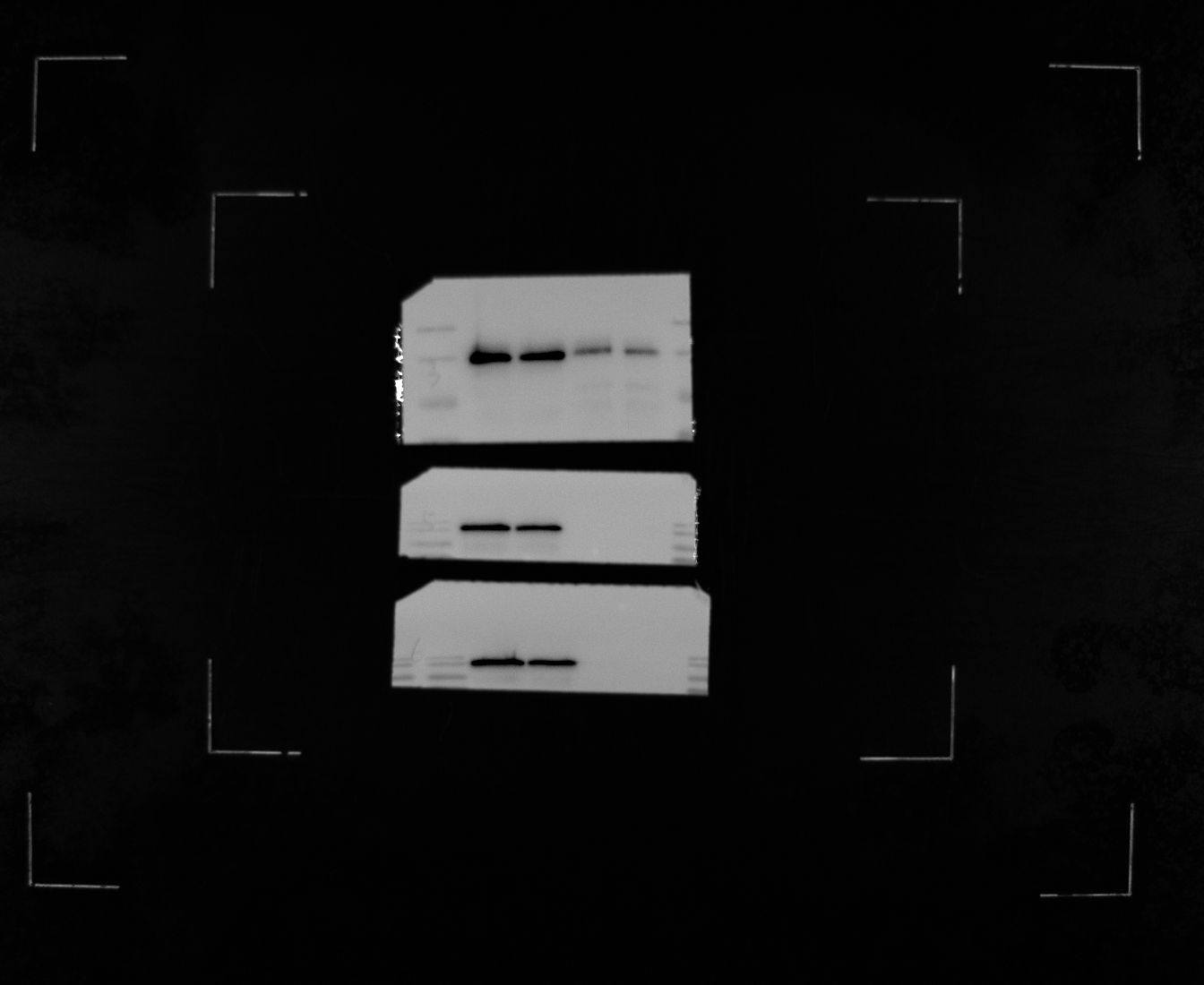

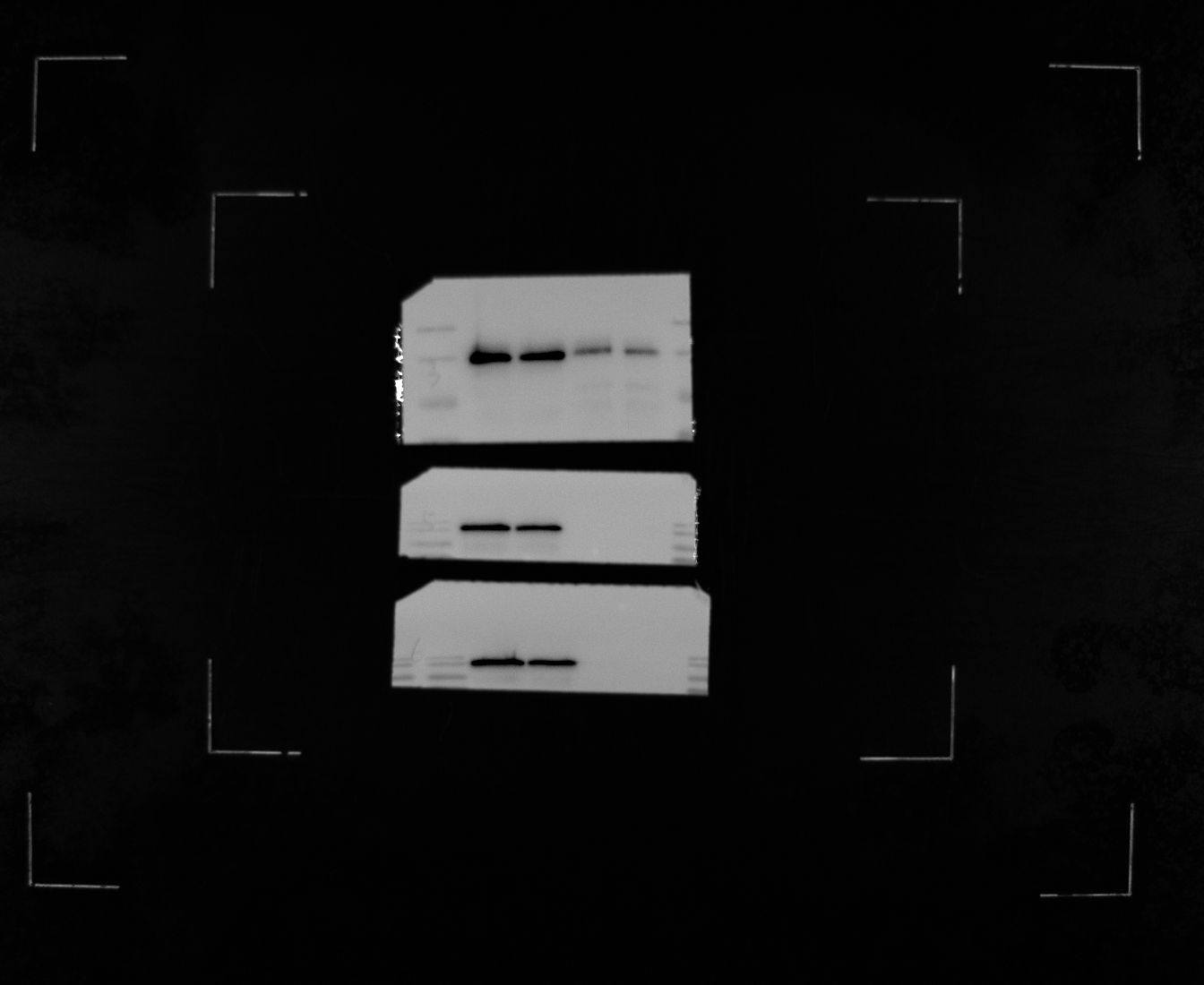

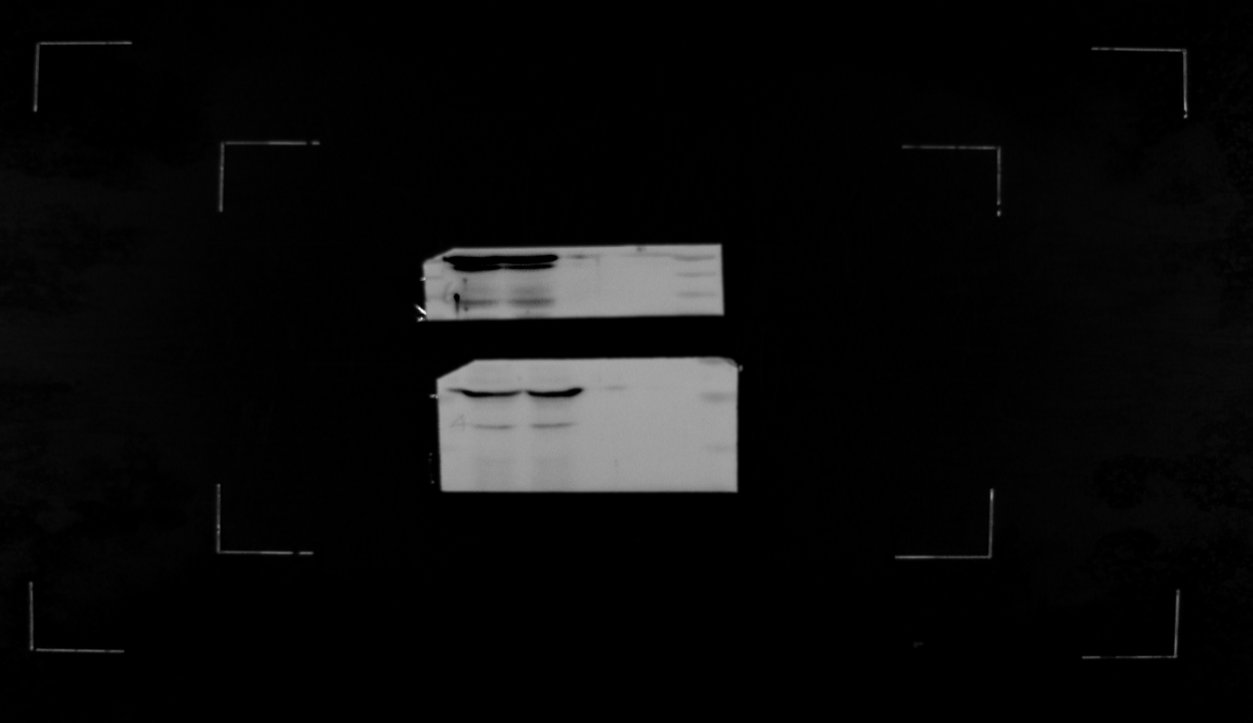

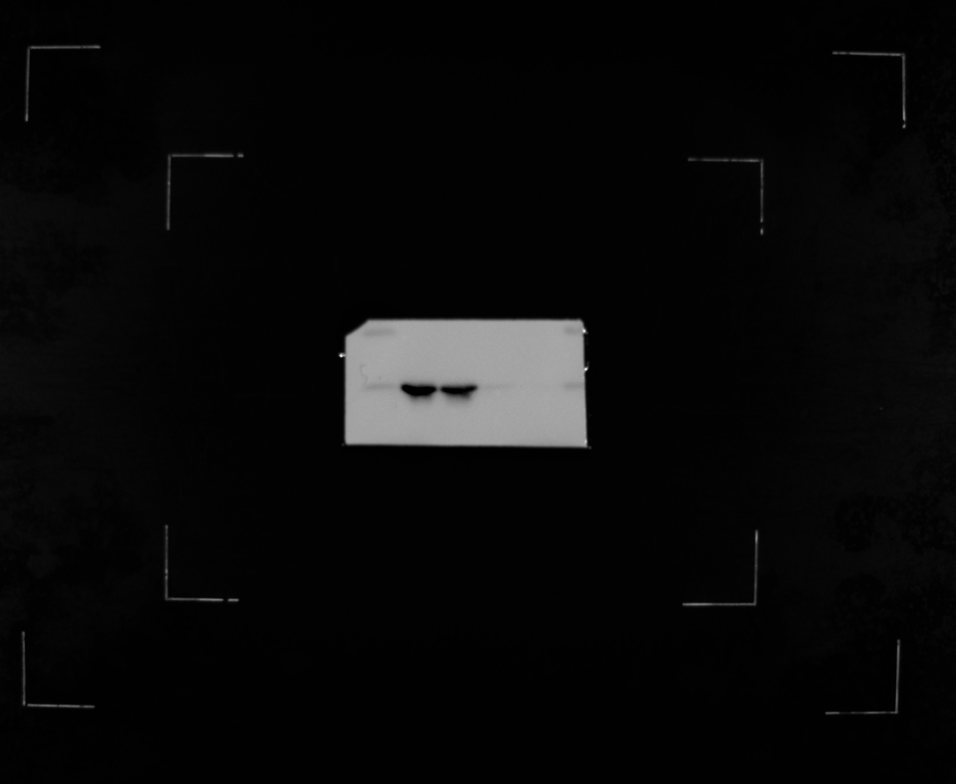

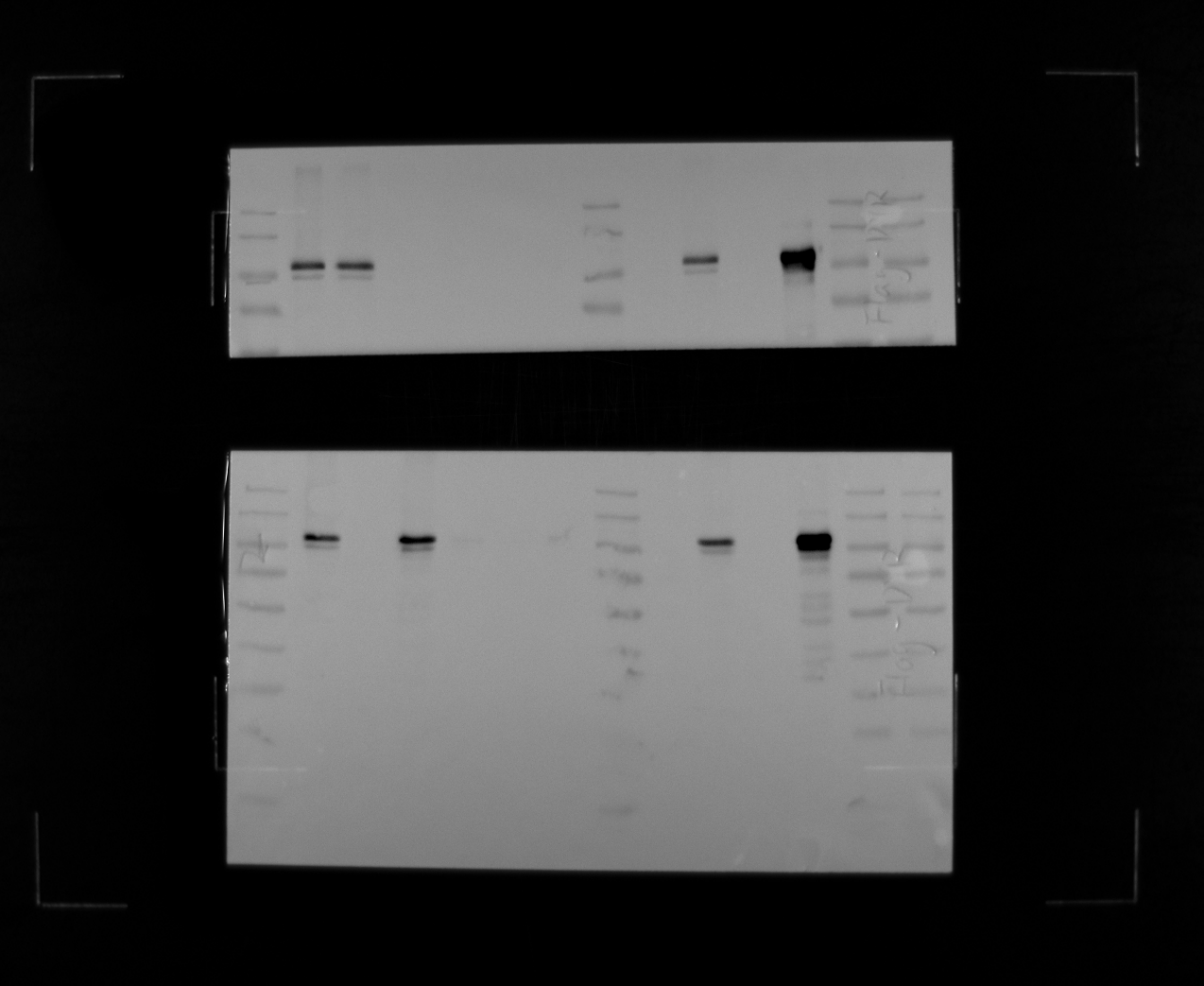

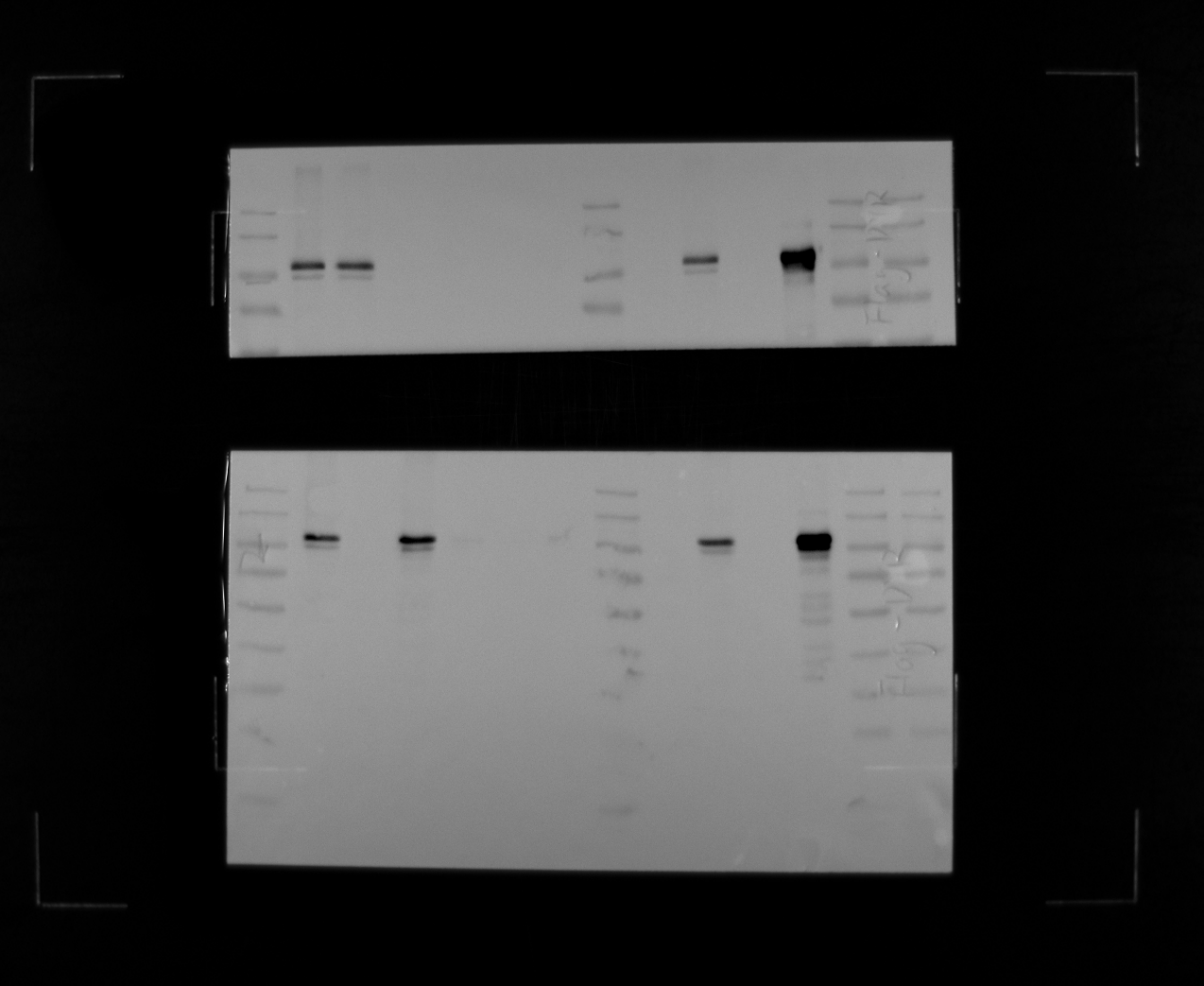


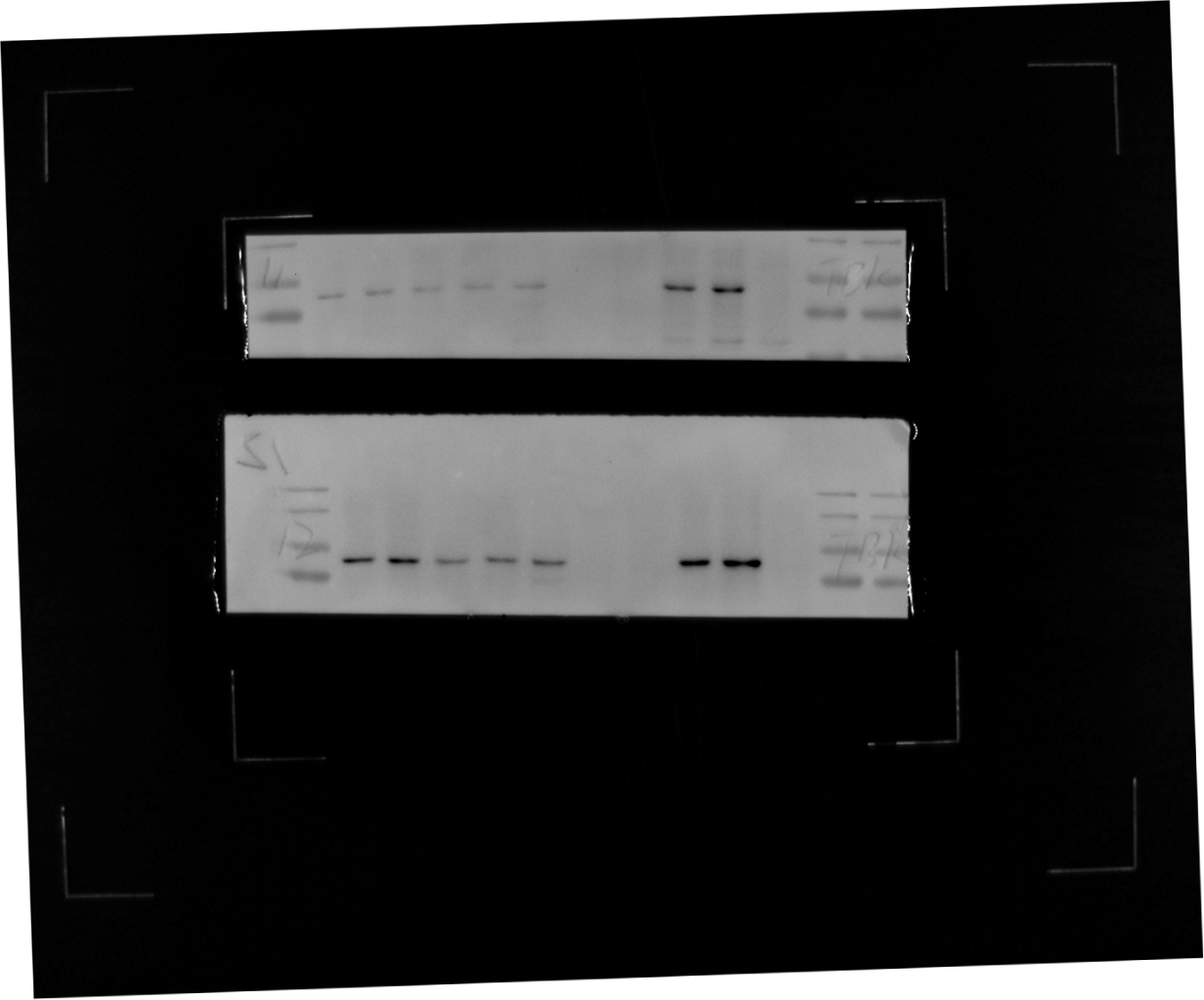

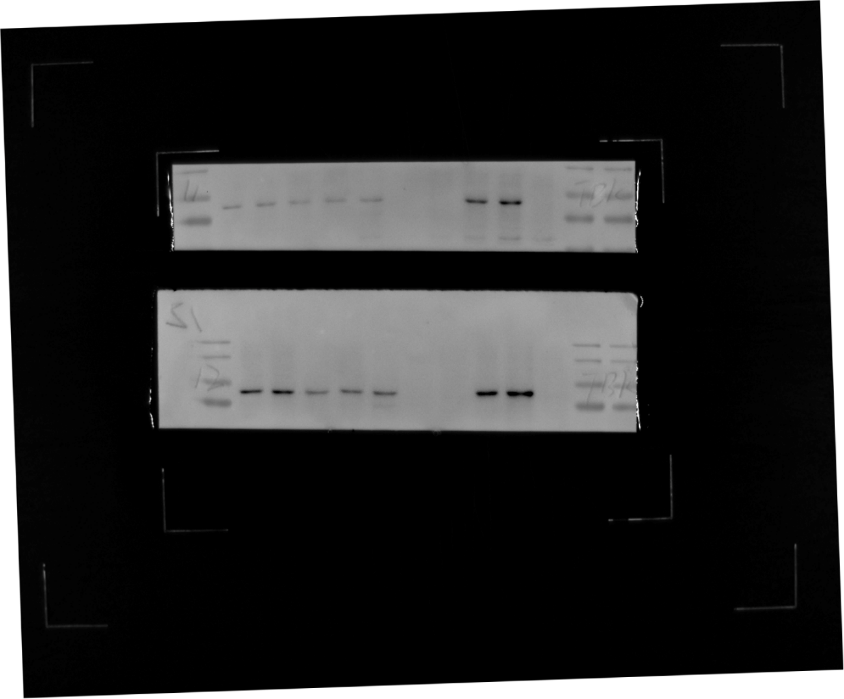


Figure 9a


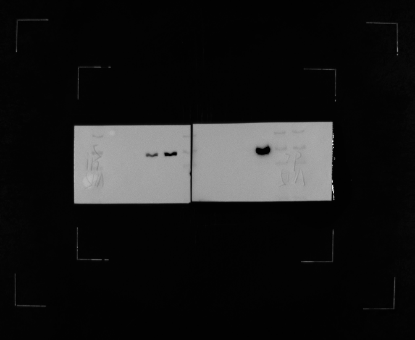


Figure S8


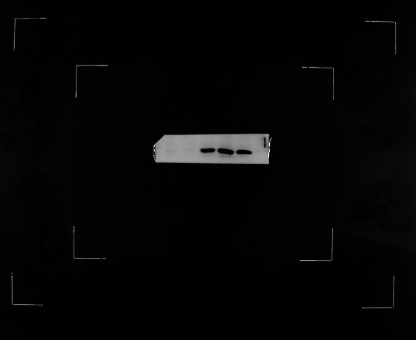

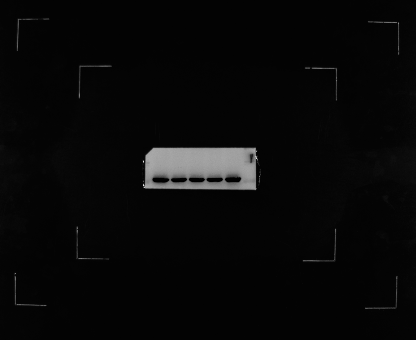

Supplement: Supplementary file 5 — Supplementary Material 5. [file 43556_2024_231_MOESM5_ESM.docx]
